# Stable polefinding and rational least-squares fitting via eigenvalues

**DOI:** 10.1007/s00211-018-0948-4

**Published:** 2018-02-21

**Authors:** Shinji Ito, Yuji Nakatsukasa

**Affiliations:** 1 Graduate School of Information Science and Technology, University of Tokyo, Tokyo, 113-8656 Japan; 2 Present Address: NEC Corporation, Kanagawa, 211-8666 Japan; 3 Mathematical Institute, University of Oxford, Oxford, OX2 6GG UK

**Keywords:** 65D05 Numerical analysis, Interpolation, 65D15 Numerical analysis, Algorithms for functional approximation

## Abstract

A common way of finding the poles of a meromorphic function *f* in a domain, where an explicit expression of *f* is unknown but *f* can be evaluated at any given *z*, is to interpolate *f* by a rational function $\frac{p}{q}$ such that $r(\gamma _i)=f(\gamma _i)$ at prescribed sample points $\{\gamma _i\}_{i=1}^L$, and then find the roots of *q*. This is a two-step process and the type of the rational interpolant needs to be specified by the user. Many other algorithms for polefinding and rational interpolation (or least-squares fitting) have been proposed, but their numerical stability has remained largely unexplored. In this work we describe an algorithm with the following three features: (1) it automatically finds an appropriate type for a rational approximant, thereby allowing the user to input just the function *f*, (2) it finds the poles via a generalized eigenvalue problem of matrices constructed directly from the sampled values $f(\gamma _i)$ in a one-step fashion, and (3) it computes rational approximants $\hat{p},\hat{q}$ in a numerically stable manner, in that $(\hat{p}+\Delta p)/(\hat{q}+\Delta q)=f$ with small $\Delta p,\Delta q$ at the sample points, making it the first rational interpolation (or approximation) algorithm with guaranteed numerical stability. Our algorithm executes an implicit change of polynomial basis by the QR factorization, and allows for oversampling combined with least-squares fitting. Through experiments we illustrate the resulting accuracy and stability, which can significantly outperform existing algorithms.

## Introduction

Let *f* be a meromorphic function in a domain ${\varOmega }$, whose explicit expression is unknown but can be evaluated at any set of sample points $\{\gamma _i\}_{i=1}^L$. This paper investigates numerical algorithms for finding the poles of *f*, along with the associated problem of finding a rational approximant $r=p/q\approx f$ in ${\varOmega }$. Finding the poles of a meromorphic or rational function *f* is required in many situations, including resolvent-based eigensolvers [4, 36, 40] and the analysis of transfer functions [30, 38].

One natural way of finding the poles is to first approximate *f* in ${\varOmega }$ by a rational function $r=p/q$, then find the poles of *r*, i.e., the roots of *q*. A common approach to obtain $r\in \mathcal {R}_{m,n}$ (a rational function of type $m,n$, i.e., $r=p/q$ for polynomials *p*, *q* of degree at most $m,n$ respectively) is to interpolate *f* at $m+n+1$ points in ${\varOmega }$ (such as the unit disk), a code for which is available in the Chebfun command ratinterp [21, 35]. However, this is a two-step process; when the poles are of primary interest, explicitly forming *r* is unnecessary and can be a cause for numerical errors. Moreover, the type of the rational function is usually required as input.

In this paper we develop a polefinding algorithm ratfun that essentially involves just solving one generalized eigenvalue problem, thereby bypassing the need to form *r*. ratfun starts by finding an appropriate type for the rational approximant from the function values: roughly, it finds the pair $(m,n)$ with a smallest possible $n$ (without taking $m$ excessively large) such that $r=p/q \approx f$ holds with $r\in \mathcal {R}_{m,n}$; in Sect. 3 we make this more precise. This allows the user to input just the function *f* to obtain the poles. The rational approximant can also be obtained if necessary.

Since polefinding for $r=p/q$ boils down to rootfinding for *q*, it is inevitable that the algorithm involves an iterative process (as opposed to processes requiring finitely many operations in exact arithmetic such as a linear system), and hence it is perhaps unsurprising that we arrive at an eigenvalue problem. Our algorithm has runtime that scales cubically with the type of the rational approximant, which is comparable to some of the state-of-the-art algorithms.

A key property of ratfun is its numerical stability. To our knowledge, no previous polefinding algorithm has been proven to be numerically stable. Numerical stability here means backward stability in the sense that $\frac{\hat{p}+\Delta p}{\hat{q}+\Delta q}=f$ holds exactly at the sample points, where $\frac{\hat{p}}{\hat{q}}$ is the computed rational approximant and $\Vert \Delta p\Vert _L/\Vert \hat{p}\Vert _L,\Vert \Delta q\Vert _L/\Vert \hat{q}\Vert _L$ are *O*(*u*) where *u* is the unit roundoff (throughout we write $x=O(y)$ to mean $x\le My$ for a moderate $M>0$), and $\Vert \cdot \Vert _L$ is the vector norm of function values at the sample points $\{\gamma _i\}_{i=1}^L$, see *Notation* below. Classical algorithms such as Cauchy’s [13], Jacobi’s [26], Thiele’s continued fractions [44, Sect. 2.2.2] and Neville-type algorithms [44, Sect. 2.2.3] are known to be of little practical use due to instability [47, Ch. 26]. The more recent Chebfun’s ratinterp is based on the SVD, and combined with a degree reduction technique, ratinterp is reliable in many situations. However, as we shall see, the algorithm is still unstable when a sample point lies near a pole. Once the numerical degree is determined, the way our algorithm ratfun finds the poles is mathematically equivalent to ratinterp (and some other algorithms), but overcomes this instability by avoiding the use of the FFT and employing a diagonal scaling to attenuate the effect of an excessively large sample value $|f(\gamma _i)|$.

Another practical method is a naive SVD-based interpolation algorithm (described in Sect. 2.1), and despite its simplicity and straightforward derivation, it works surprisingly well; indeed we prove stability in the above sense for obtaining *r* when an appropriate diagonal scaling is employed. Nonetheless, it is still based on a two-step approach, and the detour of forming the coefficients of *p*, *q* before computing the poles incurs unnecessary inaccuracy. As is well known, in rootfinding problems the choice of the polynomial basis is critical for accurate computation [47, App. 6], as Wilkinson famously illustrated in [52]. ratfun, by contrast, bypasses the coefficients and implicitly performs an appropriate change of polynomial basis.

Also worth mentioning are polefinding algorithms based on a Hankel generalized eigenvalue constructed via evaluating discretized contour integrals of the form $s_j: = \frac{1}{2\pi \mathrm {i}}\oint _\gamma z^j\frac{1}{f(z)}dz$ [29, 40]. This algorithm still has a two-step flavor (computing integrals and solving eigenproblem), and it was recently shown [3] to be mathematically equivalent to rational interpolation followed by polefinding, as in Chebfun’s ratinterp. We shall see that this algorithm is also unstable.

We shall see that ratfun is also equivalent mathematically to these two algorithms, in that our eigenproblem can be reduced to the Hankel eigenproblem by a left equivalence transformation. However, numerically they are very different, and we explain why ratfun is stable while others are not.

The contributions of this paper can be summarized as follows.

1. Polefinding (and rootfinding if necessary) by a one-step eigenvalue problem.
2. Automatic determination of a type $(m,n)$ for the rational approximant. This allows the user to obtain *p*, *q* from the input *f* alone. In previous algorithms the type $(m,n)$ has been a required input.
3. Stability analysis. We introduce a natural measure of numerical stability for rational interpolation, and establish that our algorithm ratfun is numerically stable.

Table 1 compares algorithms for polefinding and indicates the stability and complexity of each method, along with the dominant computational operation. Here, RKFIT refers to the recent algorithm by Berljafa and Güttel [8], Hankel is the algorithm based on contour integration, resulting in a generalized eigenvalue problem involving Hankel matrices (summarized in Sect. 4.8), and naive is the naive method presented in Sect. 2.1. By “avoid roots(q)” we mean the algorithm can compute the poles without forming the polynomial *q* and then finding its roots.

**Table 1:** Comparison between polefinding algorithms

	ratinterp	RKFIT	Hankel	Naive	ratfun
*p*, *q* stability	$\times$	$\times$	−	$\surd$	$\surd$
Avoid roots(q)	$\times$	$\times$	$\surd$	$\times$	$\surd$
Complexity	$O(Ln^2)$	$O(Ln^2)$	$O(n^3)$	$O(L(m+n)^2)$	$O(L(m+n)^2)$
Main computation	SVD etc	Krylov	GEP	SVD	Rectangular GEP

*GEP* generalized eigenvalue problem

This paper is organized as follows. In Sect. 2.1 we review some previous algorithms, which also leads naturally to our proposed algorithm. In Sect. 3 we discuss the process of finding an appropriate type of the rational approximation. Section 4 is the main part where our eigenvalue-based algorithm is derived, and we prove its numerical stability in Sect. 5. We present numerical experiments in Sect. 6.

*Notation* $\mathcal {P}_n$ is the set of polynomials of degree at most $n$, and $\mathcal {R}_{m,n}$ is the set of rational functions of type at most $(m,n)$. Unless mentioned otherwise, *f* is assumed to be meromorphic in a region ${\varOmega }$ in the complex plane, and $(m,n)$ denotes the type of the rational approximant $r=p/q \approx f$ that our algorithm finds: $r\in \mathcal {R}_{m,n}$, that is, $p\in \mathcal {P}_{m}$ and $q\in \mathcal {P}_{n}$. When necessary, when *f* is rational, we denote by $(M,N)$ its exact type, that is, $f=\frac{p}{q}$ where *p*, *q* are coprime polynomials of degree $M,N$, respectively. We define $c_p=[c_{p,0},c_{p,1},c_{p,2},\ldots , c_{p,m}]^\top ,c_q = [c_{q,0},c_{q,1},c_{q,2},\ldots , c_{q,n}]^\top$ to be the vectors of their coefficients such that $p(z) = \sum _{i=0}^mc_{p,i}\phi _i(z)$ and $q(z) = \sum _{i=0}^nc_{q,i}\phi _i(z)$, in which $\{\phi _i(z)\}_{i=0}^{\max (m,n)}$ is a polynomial basis, which we take to be the monomials $\phi _i(z)=z^i$ unless otherwise mentioned. When other bases are taken we state the choice explicitly. $L$ is the number of sample points, denoted by $\{\gamma _i\}_{i=1}^{L}$, which we assume to take distinct points in ${\varOmega }\in \mathbb {C}$. $F = \text{ diag }(f(\gamma _1),\ldots ,f(\gamma _L))$ is the diagonal matrix of function values at the sample points. We also let $\varGamma = \text{ diag }(\gamma _1,\ldots ,\gamma _L)$. $\Vert \cdot \Vert _L$ denotes a norm of a function, defined via the function values at the sample points $\Vert g \Vert _L= \sqrt{\sum _{i=1}^L|g(\gamma _i)|^2}$. Computed approximants wear a hat, so for example $\hat{\xi }_i$ is a computed pole. *V* is the Vandermonde matrix generated from the $L$ sample points $(\gamma _i)_{i=1}^L$ with (*i*, *j*)-element $(V)_{ij} =\gamma _i^{j-1}$:

1.1 $$\begin{aligned} V = \begin{bmatrix} 1&\gamma _1&\cdots&\gamma _1 ^{L-1} \\ 1&\gamma _2&\cdots&\gamma _2 ^{L-1} \\ \vdots&\vdots&\ddots&\vdots \\ 1&\gamma _L&\cdots&\gamma _L^{L-1} \end{bmatrix} . \end{aligned}$$

The Vandermonde matrix and its inverse play the important role of mapping between coefficient space and value space. When a non-monomial basis $\{\phi _i(z)\}$ is used, $(V)_{ij} =\phi _{j-1}(\gamma _i)$. We denote by $V_i$ the matrix of first *i* columns of *V*. *u* denotes the unit roundoff, $u\approx 10^{-16}$ in IEEE double precision arithmetic. We write $x = \Theta (y)$ to mean $x=O(y)$ and $y=O(x)$.

## Existing methods for rational interpolation and least-squares fitting

Rational interpolation is a classical problem in numerical analysis and many algorithms have been proposed, such as those by Jacobi [26], Neville and one based on continued fractions [44, Sect. 2.2]. Here we review those that can be considered among the most practical and stable. For more information on algorithms that are not explained, we refer to [12, Ch. 5], [44, Sect. 2.2] and [37, p. 59].

We first clarify what is meant by rational interpolation and least-squares fitting.

*Rational interpolation* With sample points $\gamma _i$ for $i=1,\ldots ,L$ with $L=m+n+1$, the goal of rational interpolation is to find polynomials $p\in \mathcal {P}_m,q\in \mathcal {P}_n$ satisfying the set of $L$ equations

2.1 $$\begin{aligned} f(\gamma _i) = \frac{p(\gamma _i)}{q(\gamma _i)},\quad i = 1,\ldots ,L. \end{aligned}$$

However, as is well known [12, Ch. 5], [47, Ch. 26], (2.1) does not always have a solution $p\in \mathcal {P}_m,q\in \mathcal {P}_n$. To avoid difficulties associated with nonexistence, a numerical algorithm often starts with the linearized equation

2.2 $$\begin{aligned} f(\gamma _i)q(\gamma _i) = p(\gamma _i),\quad i = 1,\ldots ,L, \end{aligned}$$

which always has solution(s), which all correspond to the same rational function $\frac{p}{q}$. Most methods discussed in this paper work with (2.2).

*Rational least-squares fitting* When we sample *f* at more than $m+n+1$ sample points $\{\gamma _i\}_{i=1}^L$ with $L>m+n+1$, (2.2) has more equations than unknowns, and a natural approach is to find *p*, *q* such that

2.3 $$\begin{aligned} f(\gamma _i)q(\gamma _i) \approx p(\gamma _i),\quad i = 1,\ldots ,L. \end{aligned}$$

This leads to a least-squares problem. Least-squares fitting is used throughout scientific computing, and it often leads to more robust algorithms than interpolation. For example, when function values contain random errors, polynomial least-squares fitting has the benefit of reducing the variance in the outcome as compared with interpolation [14, Sect. 4.5.5].

One main message of this paper is that the precise formulation of the least-squares problem (2.3) is crucial for numerical stability. For example, the minimizers of $$ \bigg \Vert \bigg [\begin{matrix} f(\gamma _1)q(\gamma _1) - p(\gamma _1)\\ \vdots \\ f(\gamma _L)q(\gamma _L) - p(\gamma _L) \end{matrix}\bigg ]\bigg \Vert $$ and $$\bigg \Vert \bigg [\begin{matrix} f(\gamma _1) - p(\gamma _1)/q(\gamma _1)\\ \vdots \\ f(\gamma _L) - p(\gamma _L)/q(\gamma _L) \end{matrix}\bigg ]\bigg \Vert $$ are clearly different. As we describe below, our method works with $$ \bigg \Vert D\bigg [\begin{matrix} f(\gamma _1)q(\gamma _1) - p(\gamma _1)\\ \vdots \\ f(\gamma _L)q(\gamma _L) - p(\gamma _L) \end{matrix}\bigg ]\bigg \Vert $$ for an $L\times L$ diagonal matrix $D=\text{ diag }(d_i)$ chosen so that

2.4 $$\begin{aligned} d_i=\frac{\text{ median }_L|f(\gamma )|}{\max (|f(\gamma _i)|,\text{ median }_L|f(\gamma )|)}. \end{aligned}$$

Here $\text{ median }_L|f(\gamma )|$ is the median value of $\{|f(\gamma _i)|\}_{i=1}^L$. This choice is crucial for establishing numerical stability.

### Naive method

Perhaps the most straightforward, “naive” method for rational interpolation is to find the coefficients of $p(z) = \sum _{i=0}^mc_{p,i}z^i$ and $q(z) = \sum _{i=0}^nc_{q,i}z^i$ by writing out (2.2) as a matrix equation

2.5 $$\begin{aligned} FV_{n+1}c_q= V_{m+1}c_p, \end{aligned}$$

where $F = \text{ diag }(f(\gamma _1),\ldots ,f(\gamma _{L}))$ for $L=m+n+1$ and $V_{m+1},V_{n+1}$ are the first $(m+1)$ and $(n+1)$ columns of *V*, the Vandermonde matrix of size $L$ as in (1.1). To obtain (2.5), note that the (partial) Vandermonde matrices map the coefficients $c_p,c_q$ to value space (i.e., $V_{n+1}c_q = [q(\gamma _1),\ldots ,q(\gamma _L)]^\top ,V_{m+1}c_p = [p(\gamma _1),\ldots ,p(\gamma _L)]^\top$), in which “multiplication by $f(\gamma _i)$” corresponds simply to “multiplication by *F*”. Equation (2.5) is thus a matrix formulation of rational interpolation (2.2) in value space.

Solving (2.5) for $c_p,c_q$ amounts to finding a null vector of the $L\times (L+1)$ matrix

2.6 $$\begin{aligned} \bigg (C \left[ \begin{array}{c} c_q\\ -c_p \end{array}\right] :=\bigg )\quad \left[ \begin{array}{cc} FV_{n+1}&{} V_{m+1}\\ \end{array}\right] \left[ \begin{array}{c} c_q\\ -c_p \end{array}\right] =0. \end{aligned}$$

Sometimes the matrix *C* has null space of dimension larger than 1; in this case all the null vectors of *C* give the same rational function *p* / *q* [12, Sect. V.3.A].

To find the poles of *f* once $c_q$ is obtained, we find the roots of the polynomial $q = \sum _{i=0}^nc_{q,i}x^i$, for example by the companion linearization [22]. When a non-monomial polynomial basis $\{\phi _i(z)\}_{i=0}^L$ is chosen, other linearizations such as comrade and confederate are available [5, 22].

The above process (2.6) can be easily extended to the oversampled case, in which $L>m+n+1$ and the matrix *C* above is of size $L\times (m+n+2)$. In this case the matrix in (2.6) has at least as many rows as columns, and does not necessarily have a null vector. Then the task is to perform a least-squares fitting, which we do by finding the right singular vector corresponding to the smallest singular value of the matrix *C*, which for later use we state as an optimization problem:

2.7 $$\begin{aligned} \begin{aligned}&\mathop {{{\mathrm{minimize}}}}\limits _{c_p,c_q}\ \left\| \begin{bmatrix} FV_{n+1}&V_{m+1} \end{bmatrix} \begin{bmatrix} c_q\\ -c_p \end{bmatrix} \right\| _2\\&\text{ subject } \text{ to } \Vert c_p\Vert _2^2 + \Vert c_q\Vert _2^2 = 1. \end{aligned} \end{aligned}$$

Here the normalization $\Vert c_p\Vert _2^2 + \Vert c_q\Vert _2^2 = 1$ is imposed to rule out the trivial solution $c_p=0,c_q=0$.

We shall consider a scaled formulation of (2.7), which left-multiplies a suitably chosen diagonal matrix *D* by the matrix in the objective function as

2.8 $$\begin{aligned} \begin{aligned}&\mathop {{{\mathrm{minimize}}}}\limits _{c_p,c_q}\ \left\| D\begin{bmatrix} FV_{n+1}&V_{m+1} \end{bmatrix} \begin{bmatrix} c_q\\ -c_p \end{bmatrix} \right\| _2\\&\text{ subject } \text{ to } \Vert c_p\Vert _2^2 + \Vert c_q\Vert _2^2 = 1. \end{aligned} \end{aligned}$$

Note that (2.7) and (2.8) have the same solution when the optimal objective value is zero, but otherwise they are different, and in the oversampled case $L>m+n+1$ this is usually the case. Numerically, they are vastly different even when $L=m+n+1$.

The dominant cost is in the SVD (more precisely computing the right singular vector corresponding to the smallest singular value) of $$\begin{bmatrix}FV_{n+1}&V_{m+1} \end{bmatrix}$$ or the scaled matrix $$D\begin{bmatrix}FV_{n+1}&V_{m+1} \end{bmatrix}$$, requiring $O(L(m+n)^2)$ cost.

The naive method (2.5) is mentioned for example in [10], but seems to be rarely used in practice, and we are unaware of previous work that explicitly investigate the least-squares formulation (2.8) or its scaled variant (2.7). Nonetheless, in Sect. 5 we shall show that the scaled formulation (2.8) is numerically stable for rational interpolation (i.e., computing *p*, *q*) for a suitable choice of *D*. In this paper we refer to (2.8) as the *scaled naive method* (or just naive method).

Another method that relies on finding a null vector of a matrix is described in [41], whose matrix elements are defined via the divided differences. Analyzing stability for this method appears to be complicated and is an open problem.

### Chebfun’s ratinterp

Chebfun [18] is a MATLAB package for working with functions based primarily on polynomial interpolation, but it also provides basic routines for rational functions. In particular, the ratinterp command runs a rational interpolation or least-squares fitting algorithm for the linearized equation (2.3), as outlined below.

We start again with the matrix equation in the naive method (2.6), which we rewrite as $FV_{n+1}c_q = V_{m+1}c_p$. Expanding the matrices $V_{m+1},V_{n+1}$ to form a full Vandermonde matrix *V*, the equation becomes

2.9 $$\begin{aligned} FV \begin{bmatrix} c_q\\0 \end{bmatrix} = V \begin{bmatrix} c_p\\0 \end{bmatrix}. \end{aligned}$$

Now when the sample points are at roots of unity $\gamma _j = \exp (\frac{2\pi \mathrm {i}j}{L})$ for $j=1,\ldots ,L$, and using the monomial basis $\{z^i\}_{i=0}^{L-1}$, we can use the FFT to efficiently multiply by *V* or $V^{-1}=\frac{1}{L}V^*$ ($V^*=\bar{V}^T$ denotes that Hermitian conjugate), and left-multiplying (2.9) by $V^{-1}=\frac{1}{L}V^*$ gives

2.10 $$\begin{aligned} \frac{1}{L}V^*FV \begin{bmatrix} c_q\\0_{L-(n+1)} \end{bmatrix} = \begin{bmatrix} c_p\\0_{L-(m+1)} \end{bmatrix}. \end{aligned}$$

The multiplication by $V^{-1}$ brings the equation back to coefficient space, and so unlike the naive method (2.5) given in value space, (2.10) is a formulation of rational interpolation in coefficient space. Note that the matrix $\frac{1}{L}V^*FV$ can be formed in $\mathcal {O}(L^2\log L)$ operations using the FFT. An analogous result holds for Chebyshev points $\gamma _j = \cos (\frac{\pi (j-1)}{L-1})$ using the Chebyshev polynomial basis [6, 46, Ch. 8].

By (2.10), $c_q$ is a null vector of the bottom-left $(L-m-1)\times (n+1)$ part of $V^*FV$, which has one more column than rows in the interpolation case $L=m+n+1$. Then the task is to find $c_q$ such that

2.11 $$\begin{aligned} \widetilde{V}^*FV_{n+1}c_q=0, \end{aligned}$$

where $\widetilde{V}$ denotes the last $L-m-1$ columns of *V* (as before, $V_{n+1}$ is the first $N+1$ columns).

Again, in the oversampled case a least-squares fitting can be done by finding the smallest singular value and its right singular vector of the $(L-m-1)\times (n+1)$ matrix $\widetilde{V}^*FV_{N+1}$.

As in the naive method, ratinterp finds the poles by finding the roots of *q* via the eigenvalues of the companion (when sampled at roots of unity) or colleague (Chebyshev points) matrices.

### RKFIT

The recent work by Berljafa and Güttel [7, 8] introduces RKFIT, a toolbox for working with matrices and rational functions based on rational Krylov decompositions. Given matrices $F,A\in \mathbb {C}^{L\times L}$ and a vector $b\in \mathbb {C}^L$, RKFIT is designed to find a rational matrix approximant *r*(*A*) to *F* such that $r(A)b\approx Fb$ by solving

2.12 $$\begin{aligned} \begin{aligned}&\mathop {{{\mathrm{minimize}}}}\limits _{r\in \mathcal {R}_{m,m}}\ \left\| \tilde{D}\left( F - r(A)\right) b\right\| _2, \end{aligned} \end{aligned}$$

where $\tilde{D}\in \mathbb {C}^{L\times L}$ is an elementwise weight matrix, which the user can specify. The objective function in (2.12) is called the absolute misfit in [8]. In the special case where $F=\text{ diag }(f(\gamma _i))$, $A= \text{ diag }(\gamma _i)$ and $b=[1,\ldots ,1]^\top$, RKFIT seeks to solve the optimization problem

2.13 $$\begin{aligned} \mathop {{{\mathrm{minimize}}}}\limits _{p,q\in \mathcal {P}_{m}}\ \left\| \tilde{D} \begin{bmatrix} f(\gamma _1) - \frac{p(\gamma _1)}{q(\gamma _1)} \\ \vdots \\ f(\gamma _L) - \frac{p(\gamma _L)}{q(\gamma _L)} \\ \end{bmatrix} \right\| _2. \end{aligned}$$

RKFIT solves (2.13) by an iterative process: starting with an initial guess for poles (e.g. $\infty$) that determines a temporary $q=q_0$, form a rational Krylov decomposition and solve (2.13) over $p\in \mathcal {P}_{m}$ via computing an SVD. Using the obtained solution, RKFIT then updates the pole estimates and $q_0$, then repeats the process until convergence is achieved. See [8] for details, which shows RKFIT can deal with more general problems, for example with multiple vectors *b* and matrices *F*.

Note that (2.13) has the flavor of dealing with the original rational approximation problem (2.1) rather than the linearized version (2.2). We observe, nonetheless, that (2.13) becomes very close to (2.8) (same except for the normalization) if we take $\tilde{D}=D\text{ diag }(q(\gamma _i))$. As we discuss in Sect. 5, the choice of *D* (and hence $\tilde{D}$) is crucial for numerical stability. Indeed, RKFIT is not stable with the default parameters when used for scalar rational approximation, but the user can input an appropriate *D* (which depends on *f*) to achieve stability.

## Automatic type determination via oversampling

A significant feature of Chebfun’s polynomial approximation process for a continuous function *f* is that the numerical degree can be obtained automatically by oversampling. This allows the user to obtain the polynomial approximant by taking just the function *f* as input, without prior information on the (numerical) degree of *f*.

Specifically, when the user inputs chebfun(f) for a function handle *f*, an adaptive process is executed to find the appropriate degree: Chebfun first samples *f* at $2^s+1$ Chebyshev points $\{\cos \frac{j\pi }{2^s}\}_{j=0}^{2^s}$ for a modest integer *s*, examines the leading Chebyshev coefficients of the interpolant, and if they have not decayed sufficiently, then increments *s* by 1 to sample at twice as many points, and repeat until the leading Chebyshev coefficients decay to *O*(*u*). For details see [2]. We emphasize the important role that oversampling plays for determining the degree; the coefficient decay is observed only after *f* is sampled more than necessary to obtain the polynomial interpolant.

For rational interpolation or approximation, we argue that it is possible to determine an appropriate type for a rational approximant just as in the polynomial case by oversampling, although the process is not just to look at coefficients but rather based on the SVD of a certain matrix. Related studies exist: Antoulas and Anderson [1] find a minimum-degree interpolant in the barycentric representation by examining a so-called L$\ddot{\text{ o }}$wner matrix, given a set of sample points. Similar techniques have been used in Chebfun’s ratinterp [21] and padeapprox [20], and in RKFIT [8], which are designed for removing spurious root-pole pairs, rather than to find a type of a rational approximant.

### Type determination by oversampling and examining singular values

Suppose that we sample a rational function $f=p/q$ at sufficiently many points $\{ \gamma _j \}_{j=1}^L$, so that $L/2$ is larger than both $\deg (p),\deg (q)$. We initially take $m=n=\lfloor (L-1) /2\rfloor$ as tentative upper bounds for the degrees. Then, as in the naive method (2.6), we compute the null space of *C* (which is square or tall, corresponding to the oversampled case). In “Appendix B” we examine the rank of the matrix *C* as the integers $m,n,L$ vary, which shows that assuming *L* is taken large enough so that $L\ge \max \{ M+ n , m + N\} + 1$, (recalling that $(M,N)$ is the exact type of *f*)

1. If $m< M$ or $n< N$, then
  3.1 $$\begin{aligned} \dim \text{ null }(C) = 0. \end{aligned}$$
2. If $m\ge M$ and $n\ge N$, then
  3.2 $$\begin{aligned} \dim \text{ null }(C) = \min (m-M,n-N)+1\ge 1. \end{aligned}$$

(See [8, Thm. 3.1] for a similar result in the RKFIT setting). Note how this result gives us information about the type of a rational *f*: By the first result, if $\dim \text{ null }(C)=0$, we need to take $m,n$ larger, along with $L$. On the other hand, if $\dim \text{ null }(C)>1$, then (3.2) shows how to reduce $n$ so that there is no redundancy: $n:=n-\dim \text{ null }(C)+1$ should give us the correct $n(=N)$ provided that $m$ was set large enough. Even if $m-M<n-N$, if $\ell >1$ singular values of *C* are negligible then we reduce $n$ by $\ell -1$ and repeat the process, which will eventually give us the correct $n=N$ provided that $m>M$. Once $n=N$ is determined, we can find $m$ as the smallest integer such that the $L\times (N+1+m+1)$ matrix *C* has a null vector. $m$ can be obtained also by looking at the leading coefficients of the computed $c_p$, but we have found this SVD-based approach to be more reliable. We emphasize the important role played by oversampling, which is necessary for (3.1) and (3.2) to hold.

The above process would find the exact type in exact arithmetic if *f* is rational. In practice, *f* may not be rational, and we compute $\dim \text{ null }(C)$ numerically by the number of singular values that are smaller than a tolerance $tol=O(u)$ to find a “numerical type” of *f*, which is the type $(m,n)$ of a rational function $r\in \mathcal {R}_{m,n}$ such that $r\approx f$ in ${\varOmega }$. It is worth noting that “numerical type” is an ambiguous notion: for example, (1) $r_1\in \mathcal {R}_{20,5}$ and $r_2\in \mathcal {R}_{5,20}$ may be equally good as approximants to *f* in the domain ${\varOmega }$, and (2) if *f* is analytic in ${\varOmega }$, polynomials would suffice if the degree is taken large enough, but rational functions give much better approximants if singularities lie near ${\varOmega }$, see [3, Sect. 6]. (1) Suggests that the “smallest” $m,n$ is not uniquely defined without further restriction. A natural approach is to find an approximant with the smallest possible $n$ (since we do not want unnecessary poles), but (2) suggests that this may lead to an approximant *p* / *q* of excessively high $\deg (p)$.

Given *f*, we attempt to find a rational approximant with as few poles as possible, within a controlled amount of computational effort. Specifically, our Algorithm 3.1 below finds a rational approximant *p* / *q* of type $(m,n)$ with the following properties:

1. There exists $p/q\in \mathcal {R}_{m,n}$ such that $\Vert fq - p\Vert _L\le tol$ and $$\left\| \big [ \begin{matrix} c_p\\ c_q \end{matrix} \big ]\right\| _2=1$$, and
2. No rational function $r=\frac{p}{q}\in \mathcal {R}_{\tilde{m},\tilde{n}}$ with $\tilde{n}< n$ and $\tilde{m}\le \lfloor (L-1)/2\rfloor$ ($\in [\max (m,n),2\max (m,n)]$) satisfies $\Vert fq - p\Vert _L\le tol$ and $$\left\| \big [ \begin{matrix} c_p\\ c_q \end{matrix} \big ]\right\| _2=1$$.

In other words, no rational function with lower denominator degree is a good approximant unless the numerator degree is more than doubled. In what follows, we set $tol=10^{-14}$ unless otherwise mentioned.

Numerically in practice, we shall show in Sect. 4.2 that it is important that a preprocessing step is carried out before examining the singular values of *C*. Specifically, we first scale *f* as $f\leftarrow f/\text{ median }_L|f(\gamma )|$ so that the median of the scaled *f* is 1 in absolute value, and left-multiply a diagonal matrix so that each row of *C* has roughly the same norm:

3.3 $$\begin{aligned} DC = \text{ diag } \left( \frac{1}{\max (|f(\gamma _i)|,1)}\right) C = [DFV_{n+1}\ DV_{m+1}]. \end{aligned}$$

This choice of *D* is the same as the one we use in the scaled naive method (2.8) for stability, to be justified in Sect. 5. Diagonal scaling has the effect of reducing the condition number (when ill-conditioning is caused by the entries having widely varying magnitudes rather than the rows being linearly dependent), and a simple scaling that scales the rows to have identical norms is known to be nearly optimal [16, 50]; the scaling in (3.3) achieves this approximately.

For further stability, we orthogonalize the two block columns by the “thin” QR factorizations.^1^ $DFV_{n+1} = Q_{n+1}R_{n+1}, DV_{m+1} = \tilde{Q}_{m+1}\tilde{R}_{m+1}$, where $Q_{n+1}\in \mathbb {C}^{L\times (n+1)},\tilde{Q}_{m+1}\in \mathbb {C}^{L\times (m+1)}$. Then we define

3.4 $$\begin{aligned} \tilde{C} = [Q_{n+1}\ \tilde{Q}_{m+1}] \end{aligned}$$

and determine the rational function type by the singular values of $\tilde{C}$. Note that (3.2) continues to hold with *C* replaced with $\tilde{C}$ in exact arithmetic.

Summarizing, Algorithm 3.1 is the pseudocode for our type determination algorithm. 
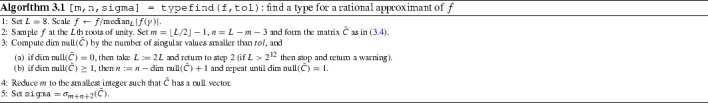


In Algorithm 3.1 we increase the number of sample points by a factor 2 until $\tilde{C}$ has a nontrivial null vector. Doubling the points allows us to reuse the previously sampled values $f(\gamma _i)$ when $\gamma _i$ are roots of unity; for the same reason, when sampling at Chebyshev points on $[-1,1]$ (this variant replaces the *L*th roots of unity in step 2 by *L* Chebyshev points), we sample at $2^s+1$ points as in Chebfun.

We note that (3.1) and (3.2) assume that sufficiently many sample points are taken so that $L\ge \max \{ M+ n , m + N\} + 1$. If this does not hold, it is possible that $\dim \text{ null }(C)>0$ although $m< M$ or $n< N$, causing Algorithm 3.1 to wrongly conclude *f* is of a lower type. Fortunately, even if $L< \max \{ M+ n , m + N\} + 1$, it is unlikely that $\dim \text{ null }(C)>0$, as this requires that $fq\approx p$ at $L$ points, where *p*, *q* together have $<L$ degrees of freedom. Similarly, a tall rectangular matrix is unlikely to have nontrivial null vectors: a random rectangular matrix is full rank with probability one, and well conditioned with high probability if the aspect ratio is safely above 1 [39]. The default value $n= L-m-3$ was chosen to ensure $\tilde{C}$ is always tall rectangular.

The description of Step 3(b) is not necessarily the most efficient: we can instead take $m\leftarrow m-\ell$ for some $\ell (>\dim \text{ null }(\tilde{C})-1)$, if this results in $\dim \text{ null }(C)>0$. In step 4, we can use bisection to determine the smallest integer *m*. The worst-case cost is thus computing $O(\log _2m)$ SVDs.

When the evaluation of *f* incurs nonnegligible (relative) error, *tol* should be adjusted accordingly. The output $\mathtt{sigma}$ indicates the error bound; a successful degree detection implies $\mathtt{sigma}<tol$.

Mathematically in exact arithmetic, the matrix *C* or $\tilde{C}$ having null space of dimension greater than 1 indicates the presence of a spurious root-pole pair, and in fact the coefficients of *p*, *q* obtained from *any* null vector of *C* are known to result in the same rational function *p* / *q*. In finite precision arithmetic, however, this property gets lost and a numerical null vector gives a function *p* / *q* that may be far from the function *f*. Furthermore, the accuracy of a computed singular vector is known to be inversely proportional to the gap between the corresponding singular value and the rest [43, Ch. 5]. Besides making the solution unique, finding the smallest possible degree has the additional benefit of widening the distance between the smallest and the second smallest singular values.

### Experiments with oversampling for degree determination

Here we illustrate typefind through some numerical experiments. For illustration purposes, instead of doubling $L=2^3,2^4,2^5,\ldots$ as in Algorithm 3.1, we formed $\tilde{C}$ for each integer $L=2,3,\ldots$ with $\gamma _j=\exp (\frac{2\pi \mathrm {i}j}{L})$, and examined the resulting output type without doubling $L$. For convenience, below we refer to this process as typefind(f,tol,L), where the number of sample points *L* is an input. 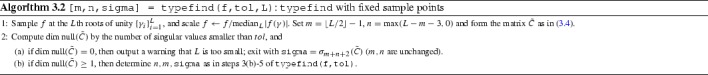


*When* *f* *is a rational function* We first examine the simplest case where *f* is a rational function

**Fig. 1:**
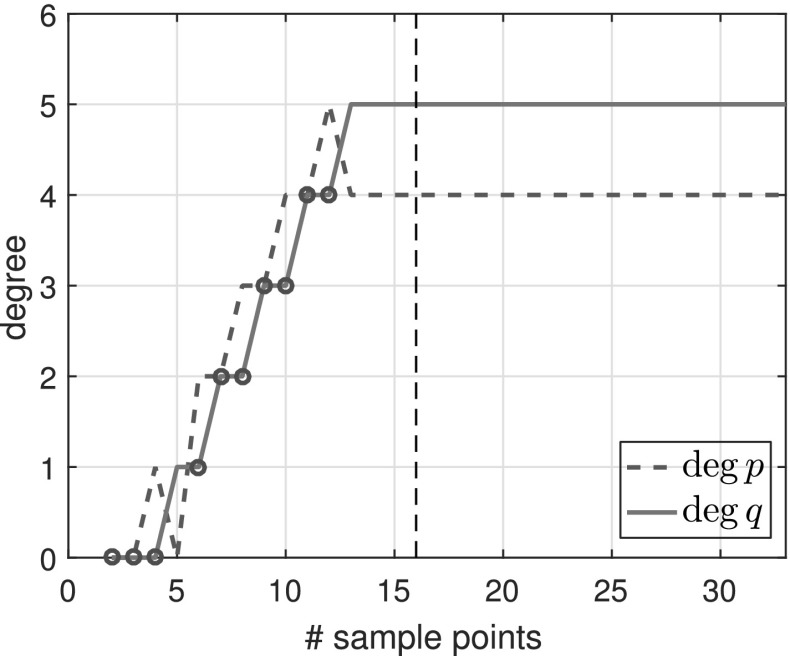
Types of the rational approximants found by typefind(f,tol,L) for rational function (3.5), as the number of sample points *L* is varied (throughout, $tol=10^{-14}$). The red circles indicate that typefind(f,tol,L) found that the number of sample points is insufficient. The vertical black dashed line indicates the number of sampled points $L$ taken by the automatic degree determination process typefind(f,tol); here $L=16$

3.5 $$\begin{aligned} f(z) = \sum _{i=1}^{N}\frac{1}{z-\xi _i} = \frac{Nz^{N-1}}{z^N- 0.9^N}, \end{aligned}$$

where $N=5$ and $\xi _i=0.9\exp (2\pi \mathrm {i} i/N)$ are equispaced on the circle of radius 0.9 centered at the origin. *f* is a rational function of exact type $(M,N) = (4,5)$. Figure 1 shows the types obtained by typefind(f,tol,L) as we increase the number of sample points *L*.

Observe that with 13 sample points or more, the algorithm correctly finds the type (4, 5) of the rational function *f*. With five sample points, however, typefind(f,tol,L) erroneously concludes that the function is of lower type; this is an artifact of the symmetry of the function (which disappears e.g. by changing the location of one pole), and illustrates the importance of oversampling. We will come back to this issue in Fig. 5.

Algorithm 3.1 samples at $2^4=16$ points to determine the type of the rational approximant. Although 16 is larger than the smallest number $M+N+1 = 10$ of sample points to theoretically obtain the rational interpolant *p* / *q* *if* the degree were known, we believe this is a small price to pay for an automated degree-finding algorithm.^2^

*When* *f* *is a meromorphic function* The situation becomes more complicated when *f* is not rational but merely meromorphic. For example consider

3.6 $$\begin{aligned} f(z) = \frac{\text{ exp }(z)}{z-\xi _1}+ \sum _{i=2}^{N}\frac{1}{z-\xi _i} . \end{aligned}$$

We take an example again with $N=5$. *f* can be regarded as being of numerical type $(\hat{M},5)$ where $\hat{M}\approx 20$, because the exponential function can be resolved to *O*(*u*) accuracy by a degree $\approx 15$ polynomial in the unit disk. Moreover, we expect that by increasing the denominator degree one can reduce the numerator degree for the same approximation quality, so we could also approximate *f* in the unit disk by a type $(20-\delta _{M} ,5+\delta _{N})$ rational function where $\delta _{M},\delta _{N}$ are modest integers such as 1, 2.

**Fig. 2:**
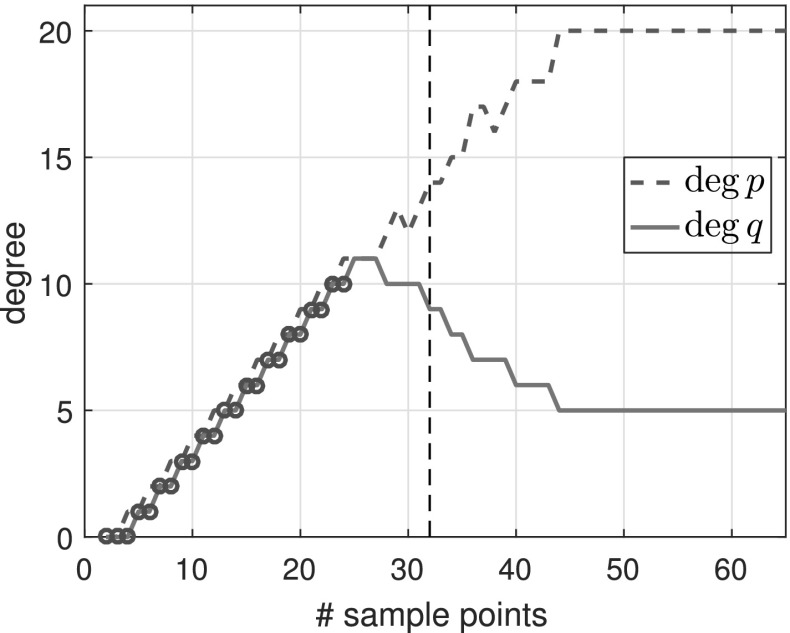
Type found by typefind(f,tol,L) for a meromorphic function *f* (3.6)

Figure 2 shows the numerical degrees obtained by typefind(f,tol,L), which confirms this observation. Algorithm 3.1 (i.e., typefind(f,tol)) outputs the type $(m,n)=(14,9)$ by sampling at $2^5=32$ points. Our polefinder ratfun (described in Sect. 4) computes nine poles, five of which approximate the correct poles $\xi _i$ to within $10^{-14}$ and four of which have absolute value larger than 10. The same is true of all the types found by $\mathtt{typefind(f,tol,L)}$ for $L\ge 25$; this example suggests they are all appropriate types, illustrating the nonunique nature of the numerical type.

*When* *f* *is an analytic function with poles near* ${\varOmega }$ Finally, we consider the function

**Fig. 3:**
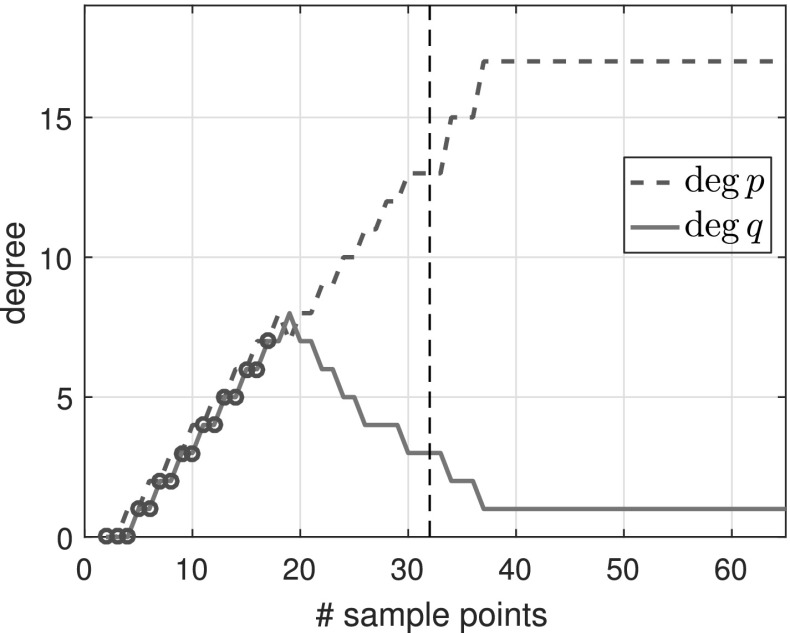
Types found by typefind(f,tol,L) for the function *f* in (3.7), analytic in the unit disk

3.7 $$\begin{aligned} f(z) = \frac{\text{ exp }(z)}{z-1.1}, \end{aligned}$$

which is analytic in the unit disk ${\varOmega }$, therefore a polynomial *p* exists such that $\Vert f-p\Vert _{\varOmega } <\epsilon$ for any $\epsilon >0$. However, as described in [3, Sect. 6], rational functions do a much better job of approximating analytic functions with a singularity lying near ${\varOmega }$, and (3.7) is such an example. Indeed, to achieve $\Vert f-p\Vert _{\varOmega } <\epsilon$ for a polynomial *p*, we need $\deg (p)\ge 386$, whereas with rationals, $\Vert f-p/q\Vert _{\varOmega } <\epsilon$ is achieved for a $p/q\in \mathcal {R}_{16,1}$. Figure 3 shows the types obtained by typefind(f,tol,L), which outputs the type (16, 1) for $L>36$. The output would become $(\deg (p),0)$ for $\deg (p)\approx 400$ if we take $L\ge 800$, but typefind(f,tol) terminates doubling the sample points once $\dim \text{ null }(\tilde{C})\ge 1$ with $L=32$, giving type (13, 3). Again, the two extra poles are far outside the unit disk.

See Fig. 7 for a function with exact poles far from the unit disk, along with other experiments in Sect. 6.

### Interpretations as optimization problems

We have emphasized the role of the diagonal scaling *D* in the discussion in Sect. 3.1. Here we reassess its significance from the viewpoint of optimization problems. Let us consider the meaning of the smallest singular value $\sigma _{m+n+2}(C)$ of *C* in (2.6), allowing for the oversampled case. As discussed in (2.7), it has the characterization

3.8 $$\begin{aligned} \sigma _{m+n+2}(C) = \min \left\{ \left\| C \begin{bmatrix} c_q \\ -c_p \end{bmatrix} \right\| _2 : \left\| \begin{bmatrix} c_q \\ -c_p \end{bmatrix} \right\| _2 =1 \right\} , \end{aligned}$$

and the solution $$\big [ \begin{matrix} c_q\\ -c_p \end{matrix} \big ] $$ is obtained by the corresponding right singular vector. Since we have $$C \big [ \begin{matrix} c_q\\ -c_p \end{matrix} \big ]= [ f(\gamma _j) q(\gamma _j)-p(\gamma _j) ]_{j=1}^L$$ by the definition of *C*, its smallest singular value $\sigma _{m+n+2}$ is equal to the optimal value of the following optimization problem:

3.9 $$\begin{aligned} \begin{aligned} \mathop {{{\mathrm{minimize}}}}\limits _{p,q}&\quad \Vert f q-p \Vert _{L} \\ \mathrm {subject\ to}&\quad p \in \mathcal {P}_{m}, \quad q \in \mathcal {P}_n, \quad \quad \Vert { c}_{p} \Vert _2^2 + \Vert { c}_{q} \Vert _2^2 = 1, \end{aligned} \end{aligned}$$

where $\Vert g \Vert _L: = \sqrt{\sum _{i=1}^L|g(\gamma _i)|^2}$. Note that the constraint in (3.9) changes depending on the choice of the polynomial basis, and the norm in the constraint $\Vert \cdot \Vert _2$ differs from that of the objective function $\Vert \cdot \Vert _L$.

Recall that for stability, instead of *C*, we work with the scaled-orthogonalized matrix $\tilde{C}$ in (3.4). We claim that the smallest singular value $\sigma _{m+n+2}(\tilde{C})$ of $\tilde{C}$ is equal to the optimal value of the following optimization problem:

3.10 $$\begin{aligned} \begin{aligned} \mathop {{{\mathrm{minimize}}}}\limits _{p,q}&\quad \Vert d f q -d p\Vert _{L} \\ \mathrm {subject\ to}&\quad p \in \mathcal {P}_{m}, \quad q \in \mathcal {P}_n,\quad \quad \Vert d p \Vert _L^2 + \Vert d fq \Vert _L^2 = 1, \end{aligned} \end{aligned}$$

where *d*(*z*) is a function such that $d(\gamma _i)$ is equal to the *i*th diagonal element of *D*.

To verify the claim, we express $\sigma _{m+n+2}(\tilde{C})$ as

3.11 $$\begin{aligned} \sigma _{m+n+2}(\tilde{C})&= \min \{ \Vert \tilde{C} x \Vert _2 : \Vert x \Vert _2 = 1 \} \\&= \min \{ \Vert Q_{ m+1 } x_2 - Q_{n+1} x_1 \Vert _2 : \Vert x_1 \Vert _2^2 + \Vert x_2 \Vert _2^2 = 1 \} \nonumber \\&= \min \{ \Vert Q_{ m+1 } x_2 - Q_{n+1} x_1 \Vert _2 : \Vert Q_{n+1} x_1 \Vert _2^2 + \Vert Q_{m+1} x_2 \Vert _2^2 = 1 \}, \nonumber \end{aligned}$$

where the last equality comes from the orthonormality of the columns of $Q_{n+1}$ and $Q_{m+1}$. From the definition of $Q_{n+1}$ and $Q_{m+1}$, we have

$$\begin{aligned} \text{ range }(Q_{n+1})&= \{ [ d( \gamma _j ) f( \gamma _j ) q( \gamma _j ) ]_{j=1} ^L: q \in \mathcal {P}_n\},&\\ \text{ range }(Q_{m+1})&= \{ [ d( \gamma _j ) p( \gamma _j ) ]_{j=1} ^L: p \in \mathcal {P}_m\}.&\end{aligned}$$

Hence, $\sigma _{m+n+2}(\tilde{C})$ in (3.11) is equal to the optimal value of the problem given by (3.10). We note that, if the optimal value $\sigma _{m+n+2}(\tilde{C})$ is sufficiently small, then the optimal solutions *p* and *q* are scaled so that $\Vert d p \Vert _L^2 \approx \Vert d f q \Vert _L^2 \approx 1/ 2$, because $|\Vert d p \Vert _L-\Vert d fq \Vert _L| \le \Vert dp-dfq\Vert _L= \sigma _{m+n+2}(\tilde{C})$.

We can also show similarly (and more easily) for the scaled (but not orthogonalized) matrix *DC* in (3.3) that $\sigma _{\min }(DC)$ is equal to the optimal value of

3.12 $$\begin{aligned} \begin{aligned} \mathop {{{\mathrm{minimize}}}}\limits _{p,q}&\quad \Vert d f q -d p\Vert _{L} \\ \mathrm {subject\ to}&\quad p \in \mathcal {P}_{m}, \quad q \in \mathcal {P}_n,\quad \quad \Vert c_p \Vert _2^2 + \Vert c_q \Vert _2 ^2 = 1. \end{aligned} \end{aligned}$$

The optimization problems (3.9), (3.10) and (3.12) differ in the following respects:

- The objective function in (3.10) and (3.12) is scaled so that $\Vert [ d(\gamma _j), d(\gamma _j) f(\gamma _j) ] \Vert _2= \Theta (1)$.
- The constraint in (3.10) does not depend on the choice of the polynomial basis.
- In (3.10), the objective function and constraint employ the same norm $\Vert \cdot \Vert _L$.

The diagonal scaling in the objective function is crucial for numerical stability as we show in Sect. 5. The independence of $\tilde{C}$ from the polynomial basis is due to the QR factorization, and it “automatically” chooses polynomial bases $\{\phi _{p,i}\}_{i=0}^{L-1}$ and $\{\phi _{q,i}\}_{i=0}^{L-1}$ for *p* and *q* respectively, for which discrete orthonormality is achieved: for *p*, defining $$v_i:=\bigg [ \begin{matrix} d( \gamma _1 ) \phi _{p,i}(\gamma _1) \\ \vdots \\ d( \gamma _L ) \phi _{p,i}(\gamma _L) \end{matrix} \bigg ]$$ we have orthonormality $v_i^*v_j=\delta _{i,j}$ (the Kronecker delta, $\delta _{i,j}=0$ if $i\ne j$ and $\delta _{i,i}=1$), and similarly for *q*, the vectors $$w_i:=\bigg [ \begin{matrix} d( \gamma _1 ) f( \gamma _1 ) \phi _{q,i}(\gamma _1) \\ \vdots \\ d( \gamma _L ) f( \gamma _L ) \phi _{q,i}(\gamma _L) \end{matrix} \bigg ]$$ are orthonormal $w_i^*w_j=\delta _{i,j}$. Note that the two bases for *p*, *q* are different, and they depend on the function *f* and sample points $\{\gamma _i\}_{i=1}^{L}$. Working with orthonormal matrices have numerical benefits, as we shall illustrate in Sect. 5.3. Together with the fact that the objective function and constraint are defined with respect to the same norm $\Vert \cdot \Vert _L$, this “scaled and QR’d” approach results in a natural and numerically stable interpolation. For these reasons, we argue that (3.10) is a natural way to formulate our problem.

Note, however, that the scaled naive method (2.8) works with (3.12), not (3.10). No QR factorizations is performed in (2.8), because if one uses it, the null vector of $\tilde{C}$ no longer gives the coefficients $c_p,c_q$ as in (2.8). Although we could retrieve $c_p,c_q$ by applying the inverse transformation with respect to the *R* factors in the QR factorizations, this leads to numerical instability when $FV_{n+1},V_{m+1}$ are ill-conditioned. In the next section we shall overcome this difficulty by formulating an algorithm that directly computes the poles, bypassing the coefficient vector $c_q$. The resulting algorithm ratfun essentially works with (3.10), but is immune to the difficulty associated with the change of polynomial basis.

## Polefinding via a generalized eigenvalue problem

We now describe our eigenvalue-based algorithm for finding the poles of *f*. Here we take $m,n$ as given, assumed to be obtained by Algorithm 3.1 or given as inputs.

### Formulating polefinding as an eigenproblem 

We consider finding the poles of $f(z) =\frac{p(z)}{q(z)}\in \mathcal {R}_{m,n}$, i.e., the roots of *q*(*z*). Denote the desired poles by $\xi _i$ for $i=1,\ldots ,n$.

As before we start with the linearized interpolation equation (2.2). Here we consider interpolation where $L=m+n+1$; we treat the oversampled case later in Sect. 4.3. The key idea is to make a pole $\xi _k$, the sought quantity, appear explicitly in the equation to be solved. To this end we rewrite *q*(*z*) using $\tilde{q}(z): = \frac{q(z)}{z-\xi _k}$, which is also a polynomial, as

4.1 $$\begin{aligned} q(z) = z\tilde{q}(z) - \xi _k\tilde{q}(z). \end{aligned}$$

We can then express (2.2) as

4.2 $$\begin{aligned} \gamma _if(\gamma _i)\tilde{q}(\gamma _i) - p(\gamma _i) = \xi _kf(\gamma _i)\tilde{q}(\gamma _i),\quad i = 1,\ldots ,m+n, \end{aligned}$$

which is the crucial guiding equation for our algorithm. The equations (4.2) can be written as a matrix equation using the Vandermonde matrix as

4.3 $$\begin{aligned} \varGamma FV_{n}c_{\tilde{q}}-V_{m+1}c_p=\xi _k FV_{n}c_{\tilde{q}}, \end{aligned}$$

where $\varGamma = \text{ diag }(\gamma _i)$, $c_{\tilde{q}}$ is the vector of coefficients for the polynomial $\tilde{q}$, and as before, $F=\text{ diag }(f(\gamma _i))$ and $V_i$ is the first *i* columns of the Vandermonde matrix. Just as in the naive method (2.5), we obtain (4.3) by mapping into value space using the Vandermonde matrix, then noting that in value space, “$f(\gamma _i)$-multiplication” is “*F*-multiplication” and “$\gamma _i$-multiplication” is “$\varGamma$-multiplication”. Thus (4.3) formulates rational interpolation again in value space, but now with $\xi _k$ shown explicitly.

Of course, $\xi _k$ is unknown in (4.3), and setting it as an unknown $\lambda$ we arrive at the generalized eigenvalue problem

4.4 $$\begin{aligned} \begin{bmatrix} A_1&A_2 \end{bmatrix}x =\lambda \begin{bmatrix} B_1&O \end{bmatrix}x, \end{aligned}$$

where $A_1 =F\varGamma V_{n}$, $A_2 = V_{m+1}$ and $B_1 = FV_{n}$, and *O* is the zero matrix of size $L\times (m+1)$.

Since the matrix $[B_1\ O]$ clearly has null space of dimension $m+1$, the eigenproblem (4.4) has $m+1$ eigenvalues at infinity. By construction, we expect the finite eigenvalues to contain information about the poles. The next result shows indeed that the finite eigenvalues of (4.4) are the poles of *f*.

#### Proposition 1

If $f(z) = \frac{p(z)}{q(z)} \in \mathcal {R}_{m,n}$ has $n$ poles counting multiplicities (i.e., $n= N$), then the matrix pencil $[A_1 , A_2] - \lambda [B_1 , O ]$ is regular, and its finite eigenvalues coincide with the poles of *f*.

(Proof) Since $f(z) \in \mathcal {R}_{m,n}$ has $n$ poles, *f*(*z*) has the expression

$$\begin{aligned} f(z)= \frac{p(z)}{q(z)} = \frac{ a \prod _{ j =1 }^d (z - \eta _j ) }{ \prod _{i=1}^n(z - \xi _i) } \end{aligned}$$

for some $d\le m$, where $a \ne 0$ and $\eta _j$ does not coincide with any element of $\{ \xi _1 , \ldots , \xi _n\}$ for $j=1, \ldots , d$, i.e., $\{ \eta _1 , \ldots , \eta _d \} \cap \{ \xi _1 , \ldots , \xi _n\} = \emptyset$. It suffices to show that $[A_1, A_2 ]- \lambda [B_1 , O]$ is singular if and only if $\lambda$ is one of the roots $\xi _1, \ldots , \xi _n$ of *q*. We can easily confirm the “if” part as follows. Let $\lambda =\xi _k$ for a fixed integer *k*. Defining the coefficient vectors $c_{\tilde{q}} = [c_{\tilde{q},0}, \ldots , c_{\tilde{q},n-1}]^\top$ and $c_p = [c_{p,0}, \ldots , c_{p,m} ]^\top$ such that $\frac{q(z)}{z - \xi _k} = \sum _{i=0}^{n-1} \tilde{c}_{q,i}\phi _i(z)$ and $p(z) = \sum _{i=0}^{m} c_{p,i} \phi _i(z)$, we have

$$\begin{aligned}&\left( ([A_1 , A_2 ] - \xi _k [B_1, O ]) \begin{bmatrix} c_{\tilde{q}}\\ -c_p \end{bmatrix} \right) _i&\\&\quad = f_i \gamma _i \frac{q( \gamma _i )}{ \gamma _i - \xi _k} - p (\gamma _i) - \xi _k f_i \frac{q( \gamma _i )}{ \gamma _i - \xi _k} = f_i q(\gamma _i) - p( \gamma _i ) =0&\end{aligned}$$

for $i = 1, \ldots , m+n+1$, so it follows that $([A_1, A_2 ]- \xi _k [B_1 , O])$ has a nontrivial kernel, and hence, $([A_1, A_2 ]- \xi _k [B_1 , O])$ is singular for $k= 1, \ldots , n$.

Next, for the “only if” part, suppose (4.4) holds for a nonzero $x = [ x_1, x_2 ] ^{\top } \in \mathbb {C}^{m+n+1}$ and $\lambda \in \mathbb {C}$, where we write $x_1 = [c_{0},\ldots ,c_{n-1}]$ and $x_2 = [d_0,\ldots ,d_{m}]$. Then, it suffices to show that $\lambda$ is one of the roots $\xi _1 , \ldots , \xi _n$ of *q*. Define polynomials $r_{x_1} (z)$ and $r_{x_2} (z)$ by $r_{x_1} (z) = \sum _{i=0}^{n-1} c_i z^i$ and $r_{x_2} (z) = \sum _{i=0}^{m} d_i z^i$, respectively. We shall show that $\lambda =\xi _i$ for some *i*, and that $(z-\xi _i)r_{x_1} (z)=Cq(z)$, $r_{x_2} (z)=Cp(z)$ for some nonzero scalar *C*. From (4.4), we have

$$\begin{aligned} z r_{x_1}(z) f(z) + r_{x_2} (z) = \lambda r_{x_1}(z) f(z) \end{aligned}$$

for $z = \gamma _1 , \ldots , \gamma _{m+n+1}$. Multiplying *q*(*z*) to both sides, we obtain

4.5 $$\begin{aligned} (z - \lambda ) r_{x_1}(z) p(z) + r_{x_2} (z) q(z) = 0 \end{aligned}$$

for $z = \gamma _1 , \ldots , \gamma _{m+n+1}$. Since the left-hand side of (4.5) is a polynomial of degree at most $m+n$ and take on the value 0 at $m+n+1$ distinct points, it must be the zero polynomial, i.e., (4.5) holds for arbitrary $z \in \mathbb {C}$. Hence, the polynomial $(z - \lambda ) r_{x_1}(z) p(z)$ is equal to the polynomial $- r_{x_2} (z) q(z)$. Note that these two polynomials are not the zero polynomial since $x \ne 0$. Let $\alpha _1, \ldots , \alpha _{d_1}$ be the roots of $r_{x_1}$ and $\beta _1, \ldots , \beta _{d_2}$ the roots of $r_{x_2}$. Since $(z - \lambda ) r_{x_1}(z) p(z)$ has the same roots as $- r_{x_2} (z) q(z)$, we have $\{ \lambda \} \cup \{ \alpha _1 , \ldots , \alpha _{d_1} \} \cup \{ \eta _{1}, \ldots , \eta _{d} \} = \{ \beta _1 , \ldots , \beta _{d_2} \} \cup \{ \xi _1 , \ldots , \xi _n\}$. Since $\{ \eta _1 , \ldots , \eta _d \} \cap \{ \xi _1 , \ldots , \xi _n\} = \emptyset$, we have $\{ \xi _1 , \ldots , \xi _n\} \subseteq \{ \lambda \} \cup \{ \alpha _1 , \ldots , \alpha _{d_1} \}$. Since the number $d_1$ of roots of $r_{x_1}$ is at most $n-1$, we have $\{ \xi _1 , \ldots , \xi _n\} = \{ \lambda \} \cup \{ \alpha _1 , \ldots , \alpha _{d_1} \}$, so it follows that $\lambda \in \{ \xi _1 , \ldots , \xi _n\}$.

We have thus shown that for every $\lambda$ and $x\ne 0$ such that (4.4) holds, $\lambda$ must be a pole of *f*. It hence follows that for any $\lambda \ne \xi _i$, the matrix $[A_1,A_2]-\lambda [B_1,0]$ is nonsingular, showing the matrix pencil is regular. $\square$

As shown in the proof, the eigenvectors of (4.4) have a special structure: the eigenvector corresponding to $\xi _i$ is

4.6 $$\begin{aligned} \begin{bmatrix} A_1&A_2 \end{bmatrix} \begin{bmatrix} c_{\tilde{q}} \\ -c_p \end{bmatrix} =\xi _i \begin{bmatrix} B_1&O \end{bmatrix} \begin{bmatrix} c_{\tilde{q}}\\ -c_p \end{bmatrix}. \end{aligned}$$

In the appendix we give further analysis of the eigenproblem, revealing the Kronecker canonical form. It shows in particular that the orders of the poles are equal to the multiplicities of the eigenvalues.

### Techniques for efficient and stable solution of eigenproblem

We now discuss how to solve (4.4) in practice. We employ techniques to remove undesired eigenvalues at $\infty$, and to achieve numerical stability.

*Projecting out eigenvalues at infinity* The generalized eigenvalue problem (4.4) has $n$ eigenvalues $\xi _i$ along with $m+1$ eigenvalues at infinity. These eigenvalues at infinity can be projected out easily. Let $A_2^\perp \in \mathbb {C}^{L\times (L-n)}$ be the orthogonal complement of $A_2$ such that $A_2^*A_2^\perp =0$. Then

4.7 $$\begin{aligned} (A_2^\perp )^* A_1x =\lambda (A_2^\perp )^* B_1x \end{aligned}$$

is an $n\times n$ eigenvalue problem whose eigenvalues are $\{\xi _i\}_{i=1}^n$ with corresponding eigenvectors $c_{\tilde{q}_i}$. To see this, recall (4.6) and note that (4.4) is equivalent to

4.8 $$\begin{aligned} \begin{bmatrix} A_2&A_1 \end{bmatrix} \begin{bmatrix} -c_p\\c_{\tilde{q}} \end{bmatrix} =\lambda \begin{bmatrix} O&B_1 \end{bmatrix} \begin{bmatrix} -c_p\\c_{\tilde{q}} \end{bmatrix}, \end{aligned}$$

and so taking the QR factorization $A_2 = [Q_1\ A_2^\perp ]R$, by left-multiplying $[Q_1\ A_2^\perp ]^*$ we obtain

4.9 $$\begin{aligned} \begin{bmatrix} R&Q_1^* A_1\\ O&(A_2^\perp )^* A_1 \end{bmatrix} \begin{bmatrix} -c_p\\c_{\tilde{q}} \end{bmatrix} =\lambda \begin{bmatrix} O&Q_1^*B_1\\ O&(A_2^\perp )^*B_1 \end{bmatrix} \begin{bmatrix} -c_p\\c_{\tilde{q}} \end{bmatrix} \end{aligned}$$

from which we can deflate the $m+1$ eigenvalues corresponding to the top-left corner and solve for the lower-right part, to arrive at (4.7) with eigenvector $c_{\tilde{q}}$. Alternatively, (4.8) shows that the “residual” $A_1x_1-\lambda B_1x_1\in \text{ Span }(A_2)$, which means it is orthogonal to $A_2^{\perp }$; (4.7) is its representation.

*Diagonal scaling* Generally, given an eigenvalue problem $A-\lambda B$, a well known technique of balancing the elements in the presence of widely varying elements is diagonal scaling.

As with the scaled naive method (2.8), we left-multiply a diagonal matrix *D* and work with the pencil $D(A-\lambda B)$ so that each row of $D[A\ B]$ has about the same norm. In Sect. 5 we show that this scaling makes our approach numerically stable.

*Orthogonalization* As alluded to at the end of Sect. 3.3, the final technique that we use for improved stability, which is inapplicable in the naive method, is orthogonalization. As in (3.4), we take the thin QR factorizations $DA_2=Q_{A_2}R_{A_2},DB_1=Q_{B_1}R_{B_1}$, where $Q_{A_2},Q_{B_1}$ have orthonormal columns and are of the same size as $DA_2,DB_1$. The rationale is that numerical errors are reduced by working with orthogonal matrices. These can be computed exploiting the Vandermonde structure, as explained after (3.4).

Applying scaling and orthogonalization to (4.9), the eigenvalue problem we solve becomes

4.10 $$\begin{aligned} \tilde{A} x = \lambda \tilde{B}x,\quad \text{ where }\; \tilde{A} = (Q_{A_2}^{\perp })^*\varGamma Q_{B_1}, \tilde{B}= (Q_{A_2}^{\perp })^*Q_{B_1}x. \end{aligned}$$

This is a $n\times n$ eigenproblem; the $n$ eigenvalues are precisely the $n$ sought poles.

Recall that as a consequence of this orthogonalization, the eigenvector of (4.10) goes through the change of basis with respect to $R_{B_1}^{-1}$. This severely affects the naive method (for which the singular vector is the sought quantity), but not our algorithm (for which the eigenvalues are sought).

*Use of FFT?* In the practically important cases where the sample points are at roots of unity or Chebyshev points, we can use the FFT to efficiently obtain the matrices in (4.7), as discussed in Sect. 2.2.

However, we shall not use the FFT in this work, for two reasons. First, while the FFT significantly speeds up the matrix-matrix multiplication, from $O(L^3)$ to $O(L^2\log L)$, this is not essential to the overall algorithm as it inevitably invokes an eigensolver (or an SVD), which requires $O(L(m{+}n)^2)$ operations. Indeed [35] designs the algorithm to fascilitate the use of the FFT, but again the saving is attenuated by the $O(Ln^2)$ SVD step.

The second, more fundamental, reason is stability. We shall see in Sect. 5 and through numerical experiments that diagonal scaling is crucial for stability. Unfortunately, using the FFT makes diagonal scaling inapplicable.

*Pole exactly at sample point* When a pole happens to exactly coincide with a sample point, $f(\gamma _i)=\infty$ and the eigenvalue problem breaks down due to infinity elements in the matrices. However, this should be a “happy” breakdown, rather than a difficulty. In this case we can simply take $\gamma _i$ to be a computed pole, and work with the function $f:=(z-\gamma _i)f$, taking $n:=n-1$. An alternative and equally valid approach is to take $f(\gamma _i)=\frac{1}{u}\text{ median }_L|f(\gamma )|$, and proceed as usual.

### Oversampling and least-squares fitting

As with previous algorithms, it is often recommended to take advantage of the oversampled values $f(\gamma _i)$ at more than $m+n+1$ points $\gamma _i$, and perform a least-squares fitting. This is true especially in our context, where the degree-finding process in Algorithm 3.1 has oversampled *f* to find the type, and it is natural to try to reuse the computed quantities $f(\gamma _i)$.

Consider finding the poles of $f(z) = \frac{p(z)}{q(z)} \in \mathcal {R}_{m,n}$ with $L> m+n+1$ sample points $(\gamma _i)_{i=1}^L$. We form the matrices as in the previous Sect. (4.4) with $A_1 = \varGamma F V_{n} \in \mathbb {C}^{L\times n}$, $A_2 = V_{m+1} \in \mathbb {C}^{L\times (m+1)}$ and $B_1 = FV_n\in \mathbb {C}^{L\times n}$. We proceed as in (4.10) and apply projection, scaling, and orthogonalization $DA_2=Q_{A_2}R_{A_2},DB_1=Q_{B_1}R_{B_1}$, to obtain matrices $\tilde{A} = (Q_{A_2}^{\perp })^*\varGamma Q_{B_1}, \tilde{B}=(Q_{A_2}^{\perp })^*Q_{B_1}.$ as in (4.10), but these matrices are now nonsquare, of size $(L-m-1)\times n$: they have more rows than columns since $L>m+n+1$. Under the assumption that *f* has $n$ poles, there exists a nonzero $x \in \mathbb {C}^n$ with $( \tilde{A} - \lambda \tilde{B} ) x =0$ if and only if $\lambda$ is one of the poles of *f*; this can be shown as in Proposition 1. Hence, in theory, we can compute the poles of *f* by solving the *rectangular* eigenvalue problem

4.11 $$\begin{aligned} \tilde{A}x = \lambda \tilde{B} x, \quad x \ne 0 . \end{aligned}$$

However, traditional methods for generalized eigenvalue problems such as the QZ algorithm [19, Ch. 7], [31] are not applicable to (4.11) since the pencil $\tilde{A} - \lambda \tilde{B}$ is rectangular.

To solve the rectangular eigenvalue problem (4.11), we use the recent algorithm by Ito and Murota [25]. The idea is to find perturbations $\Delta A,\Delta B$ with smallest $\Vert [\Delta \tilde{A}\ \Delta \tilde{B}]\Vert _{\mathrm {F}}$ so that the pencil $(\tilde{A}+\Delta \tilde{A}) - \lambda (\tilde{B}+\Delta \tilde{B})$ has $n$ eigenpairs:

4.12 $$\begin{aligned} (\tilde{A}+\Delta \tilde{A})[x_1,\ldots ,x_n] = (\tilde{B}+\Delta \tilde{B}) [x_1,\ldots ,x_n] \begin{bmatrix} \lambda _1&\\ {}&\ddots&\\&\lambda _n\end{bmatrix}. \end{aligned}$$

The resulting algorithm computes the SVD $$ [\tilde{A}\ \tilde{B}]= U \Sigma \big [ \begin{matrix} W_{11}^*&{} W_{21}^*\\ W_{12}^*&{} W_{22}^* \end{matrix} \big ]$$, then solves the *square* $n\times n$ generalized eigenvalue problem

4.13 $$\begin{aligned} W_{11}^*x=\lambda W_{21}^* x. \end{aligned}$$

This corresponds to taking $$\tilde{A}+\Delta \tilde{A} = U\Sigma \big [ \begin{matrix} W_{11}^*\\ O \end{matrix} \big ]$$ and $$\tilde{B}+\Delta \tilde{B} = U\Sigma \big [ \begin{matrix} W_{21}^*\\ O \end{matrix} \big ]$$, hence $\Vert [\Delta \tilde{A} \Delta \tilde{B}]\Vert _{\mathrm {F}}^2=\sum _{i=n+1}^{\min \{ 2 n, L - m - 1 \}}\sigma _{ i }([\tilde{A} \ \tilde{B}]) ^ 2$. See [25] for details.

### Pseudocode

Summarizing, the following is the pseudocode for our polefinding algorithm ratfun. 
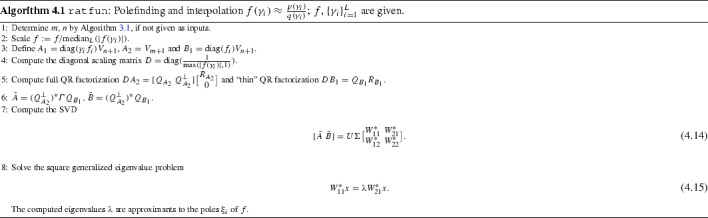
 By default, the sample points $\gamma _i$ are the roots of unity $\gamma _j = \exp (\frac{2\pi \mathrm {i}j}{L})$ (once $L$ is specified); other choices are allowed such as Chebyshev points on $[-1,1]$. We justify the scaling in step 2 and the choice of the diagonal matrix *D* in Sect. 5.2.

When the domain of interest is far from the origin, it is recommended that one work with a shifted function $f(z-z_0)$ so that the domain becomes near the origin (this affects the Vandermonde matrix, in particular its condition number).

#### Efficiency

The dominant costs of Algorithm 4.1 are in the QR factorizations, forming $Q_{A_2}^{\perp }$, computing the SVD (4.14) and solving the eigenproblem (4.15). These are all $O(L(m+n)^2)$ or less, using standard algorithms in numerical linear algebra. This is comparable in complexity with other approaches, as we summarized in Table 1.

### Input/output parameters

Combined with the degree determination process Algorithm 3.1, ratfun lets us find poles and rational interpolants with the minimum input requirement: just the function *f*. Our algorithm ratfun (described in detail in Sect. 4) adapts to the specifications as necessary when the user inputs more information such as the location of the sample points and type of the rational approximants. Below we detail the process for three types of inputs:

1. Minimum input requirement: poles = ratfun(f).
2. Function and sample points: poles = ratfun(f,gam).
3. Function, sample points and degrees: poles = ratfun(f,gam,m,n).

*Minimum input requirement* poles = ratfun(f) When the function *f* is the only input the algorithm first determines the numerical type of the rational approximant by Algorithm 3.1, then runs the polefinding algorithm to be described in Sect. 4. By default, we take the sample points to be roots of unity; Chebyshev points can be chosen by invoking ratfun(f,‘c’).

*Inputs are function and sample points* poles = ratfun(f,gam) When the sample points are specified by gam the algorithm first runs the degree finder typefind(f,tol,L) with $L=\mathtt{length(gam)}$, and gives a warning if the number of sample points $L$ appears to be insufficient $L< \max \{ M+ n , m + N\} + 1$, indicated by $\sigma _{m+n+2}(\tilde{C})>tol$. Regardless, the algorithm proceeds with solving the generalized eigenvalue problem to obtain approximate poles and *p*, *q* with $\deg (p)+\deg (q)+2\le L$. We note that the backward errors $\Vert \Delta p\Vert _L$ and $\Vert \Delta q\Vert _L$ have magnitudes $O(\sigma _{m+n+2}(\tilde{C}))$, which is not necessarily *O*(*tol*) in this case (see Sect. 5 for details on backward errors).

*Full input: function, sample points and degrees* poles = ratfun(f,gam,m,n) When the degrees are further specified the algorithm directly solves the (rectangular or square when $L=m+n+1$) generalized eigenvalue problem to obtain the poles and *p*, *q*.

#### Outputs

The full output information is [poles,cp,cq,type,roots]=ratfun(f), in which poles are the computed poles, cp,cq are the vectors of coefficients $c_p,c_q$ of the polynomials *p*, *q* in the monomial basis, type is a 2-dimensional vector $[\hat{m},\hat{n}]$ of the computed type, and roots are the computed roots.

We next discuss how to compute the roots and finding $c_p,c_q$.

### Computing the roots

One situation that Algorithm 4.1 did not deal with is when the roots of *f* are sought. We suggest two approaches for rootfinding, depending on whether poles are also sought or not.

*Finding roots only* First, when only the roots are of interest, we can invoke Algorithm 4.1 to find the poles of 1 / *f*. Alternatively, the roots can be computed from *f* by defining $\tilde{p}(z) = \frac{p(z)}{z-r_k}$ and starting from the guiding equation [recall (4.2)]

4.16 $$\begin{aligned} f(\gamma _i)q(\gamma _i) = z\tilde{p}(\gamma _i) - r_k\tilde{p}(\gamma _i), \quad i = 1,\ldots ,m+n, \end{aligned}$$

which, as before, can be rewritten as a generalized eigenvalue problem with $\lambda :=r_k$. For brevity we omit the details, as the formulation is analogous to that for (4.4) and (4.11).

*Finding poles and roots* When both the poles and roots are required, we suggest the following. First compute the poles as in Algorithm 4.1. Then we find the roots by solving for $\lambda$ the equation

4.17 $$\begin{aligned} f(\gamma _i)q(\gamma _i)=(\gamma _i-\lambda )\tilde{p}(\gamma _i), \quad \tilde{p}(z)= \frac{p(z)}{z-\lambda } . \end{aligned}$$

Here $\tilde{p}(z)$ is the same as above, and we form $q(\gamma _i)$ from the expression $q(z)=\prod _{i=1}^n(z-\hat{\xi }_i)$ using the poles $\hat{\xi }_i$ that have previously been computed. Equation (4.17) can be rearranged to $\gamma _i\tilde{p}(\gamma _i) - f(\gamma _i)q(\gamma _i)=\lambda \tilde{p}(\gamma _i)$, which we write in matrix form as

4.18 $$\begin{aligned} \begin{bmatrix} F\text{ diag }(q(\gamma _i))&\varGamma V \end{bmatrix} \begin{bmatrix} 1 \\ -c_{\tilde{p}} \end{bmatrix} =\lambda \begin{bmatrix} 0&V \end{bmatrix} \begin{bmatrix} 1 \\ -c_{\tilde{p}} \end{bmatrix}. \end{aligned}$$

This is again a rectangular generalized eigenvalue problem. This has one irrelevant eigenvalue at infinity, and the problem can again be solved via an SVD. Since the matrices involved are of smaller size than (4.4) and (4.11), this process is cheaper than finding the poles of 1 / *f*.

### Finding $c_p,c_q$

To find the coefficient vectors $c_p$ and $c_q$, we can take advantage of the eigenvector structure (4.6) to extract $c_p$ from any eigenvector, along with $c_{\tilde{q}}$, from which we obtain $c_q$ via (4.1). Note that to do this we need to give up the QR factorization in step 4 of Algorithm 4.1. Equally effective and stable is to invoke the scaled naive method (2.8), which gives $c_p,c_q$ directly (our current code adopts this approach). A word of caution is that eigenvectors are sensitive to perturbation if (but not only if) the corresponding eigenvalues are nearly multiple.

We note that there are many other ways of representing a rational function $f=p/q$. Since ratfun can compute the poles and roots as described above, one effective representation is to take

4.19 $$\begin{aligned} f = c\frac{\prod _{i=1}^m(z-\hat{r}_i)}{\prod _{i=1}^n(z-\hat{\xi }_i)} , \end{aligned}$$

in which we store the constant *c* and the roots $\hat{r}_i$ and poles $\hat{\xi }_i$.

### Mathematical equivalence with previous algorithms: interpolation-based and Hankel eigenproblem 

Here we briefly discuss the connection between our eigenvalue problem and existing ones. We shall show that the eigenproblem (4.7), when $L=m+n+1$, is equivalent in exact arithmetic to the generalized eigenvalue problem of Hankel matrices derived in [29, 40], which are in turn equivalent to Chebfun’s ratinterp as shown in [3]. Essentially, both our algorithm and ratinterp find the roots of *q* such that $\frac{p}{q}$ interpolates *f* at the sample points.

We shall show that the eigenvalues and right eigenvectors of (4.7) and those of the Hankel matrix pencil are the same. Before proving this claim, we briefly review the Hankel eigenproblem approach, which originates in work of Delves and Lyness [15] and Kravanja et al. [27, 28], see also [3]. In this algorithm, one computes the discretized moments

$$\begin{aligned} s_j: = \frac{1}{2\pi \mathrm {i}}\oint _{ |z| = 1 } z^jf(z) \mathrm {d}z, \quad j = 0,\ldots , 2n-1, \end{aligned}$$

and then solves the generalized eigenvalue problem with Hankel matrices4.20We write this as $H_1x = \lambda H_0x$ for simplicity. The pencil $H_1 - \lambda H_0$ can be written using a contour integral as

4.21 $$\begin{aligned} H_1 - \lambda H_0 = \oint _{|z| = 1} (z - \lambda ) f(z) \begin{bmatrix} 1 \\ \vdots \\ z^{n-1} \end{bmatrix} [ 1 ~ \cdots ~ z^{n-1} ] \mathrm {d} z . \end{aligned}$$

If *f* is meromorphic in the unit disk $\{ z \in \mathbb {C} \mid |z | \le 1 \}$ and has $n$ poles $\xi _1 , \ldots , \xi _n\in \{ z \in \mathbb {C} \mid |z | < 1 \}$, then the poles are eigenvalues of $H_1 x = \lambda H_0 x$. Indeed, defining $\tilde{q} = \prod _{l \ne k , 1 \le l \le n} (z - \xi _l)$ and letting $c_{\tilde{q}}$ be its coefficient vector as in (4.3) we obtain

4.22 $$\begin{aligned} (H_1 - \xi _k H_0) c_{\tilde{q}} = \oint _{|z| = 1} (z - \xi _ k) f(z) \tilde{q}(z) \begin{bmatrix} 1 \\ \vdots \\ z^{n-1} \end{bmatrix} \mathrm {d} z = 0, \end{aligned}$$

since $(z - \xi _ k) f(z) \tilde{q}(z) = f(z) \prod _{k=1}^n(z - \xi _k)$ is analytic in the unit disk.

The contour integral (4.22) needs to be discretized in a practical computation. If we use the standard trapezoidal rule evaluating at roots of unity $\gamma _j = \exp ( 2 \pi \mathrm {i}j / L)$ for $j=1,\ldots , L$ to approximate $H_1 - \lambda H_0$, the computed pencil $\hat{H}_1 - \lambda \hat{H}_{0}$ becomes

4.23 $$\begin{aligned}&\hat{H}_1 - \lambda \hat{H}_{0} \nonumber \\&\quad = \sum _{j=1}^L\frac{2 \pi \mathrm {i} \gamma _j}{L} ( \gamma _j - \lambda )f(\gamma _j) \begin{bmatrix} 1 \\ \vdots \\ \gamma _j^{n-1} \end{bmatrix} [ 1 ~ \cdots ~ \gamma _j^{n-1} ] \nonumber \\&\quad = \frac{2 \pi \mathrm {i} }{L} \begin{bmatrix} \gamma _1&\gamma _2&\cdots&\gamma _L\\ \vdots&\vdots&\vdots \\ \gamma _1 ^{n}&\gamma _2 ^{n}&\cdots&\gamma _L^{n} \end{bmatrix} \begin{bmatrix} (\gamma _1 - \lambda )f(\gamma _1 )&&\\&\ddots&\\&\ddots&\\&&(\gamma _L- \lambda ) f(\gamma _L) \end{bmatrix} \begin{bmatrix} 1&\cdots&\gamma _1 ^{n-1} \\ 1&\cdots&\gamma _2 ^{n-1} \\ \vdots&\vdots \\ 1&\cdots&\gamma _L^{n-1} \end{bmatrix} \nonumber \\&\quad = V_n^\top \varGamma ^2 F V_{n} - \lambda V_n^\top \varGamma F V_{n} , = (V_n^\top \varGamma F) (\varGamma -\lambda I) V_{n}, \end{aligned}$$

where $F = \text{ diag }(f(\gamma _1),\ldots ,f(\gamma _{L}))$ and $\varGamma = \text{ diag }(\gamma _1,\ldots ,\gamma _L)$ as before. Hence if *f* is a rational function $f=\frac{p}{q}$, we have

$$\begin{aligned} ( \hat{H}_1 - \xi _i \hat{H}_{0})c_{\tilde{q}}&=(V_n^\top \varGamma ) F(\varGamma -\xi _i I) [\tilde{q}(\gamma _1),\ldots , \tilde{q}(\gamma _L)]^\top \\&=V_n^\top \varGamma [p(\gamma _1),\ldots , p(\gamma _L)]^\top \\&=V_n^\top [\gamma _1p(\gamma _1),\ldots , \gamma _Lp(\gamma _L)]^\top . \end{aligned}$$

The *i*th element of the final vector is $\sum _{j=1}^L\gamma _j^{i+1}p(\gamma _i)$ for $i=1,\ldots ,n$, which is equal to the evaluation of $\frac{1}{2\pi \mathrm {i}}\oint _{|z|=1}^Lz^{i} p(z)$. Now the $L$-point trapezoidal rule is exact if the integrand is polynomial of degree $L-1$ or below [49, Cor. 2.3]. Therefore, if $L\ge m+n+1$ then $( \hat{H}_1 - \xi _i \hat{H}_{0})c_{\tilde{q}}=0$. Thus also for the discretized pencil $\hat{H}_1 - \xi _i \hat{H}_{0}$, $c_{\tilde{q}}$ is again an eigenvector if $f=\frac{p}{q}$ with $p\in \mathcal {P}_{m},q\in \mathcal {P}_{n}$ with $L\ge m+n+1$.

This shows that the eigenproblems $\hat{H}_1x = \lambda \hat{H}_{0}x$ and $(A_2^\perp )^* A_1x=\lambda (A_2^\perp )^*B_1x$ in (4.9) have the same eigenvalues and eigenvectors, thus are equivalent, i.e., there exists a nonsingular matrix *W* such that $W\hat{H}_1=(A_2^\perp )^* A_1$ and $W\hat{H}_{0}=(A_2^\perp )^*B_1$.

Despite the mathematical equivalence, we reiterate that the numerical behavior of the algorithms is vastly different. Crucially, the left-multiplication by $V_n^\top$ in (4.23) mixes up the magnitudes of $f(\gamma _i)$, resulting in the instability due to near-pole sampling. This will be made precise in the next section.

## Numerical stability

A crucial aspect of any numerical algorithm is stability [23]. It is common, and often inevitable for problems that are potentially ill-conditioned, to investigate backward stability (as opposed to analyzing the forward error in the outcome itself), in which we ask whether a computed output is guaranteed to be the exact solution of a slightly perturbed input.

The great success of polynomial interpolation of a continuous function *f* at roots of unity (for approximation in the unit disk) or Chebyshev points (on an interval $[-1,1]$) is due to its combined efficiency and stability: a degree-*n* polynomial interpolation can be done in $O(n\log n)$ operations employing the Chebyshev polynomials and FFT [6]. Moreover, since the FFT matrix has condition number 1, the process is numerically stable, and we obtain an interpolant $\hat{p}$ satisfying

5.1 $$\begin{aligned} f(\gamma _i) = \hat{p}(\gamma _i)+O(u\Vert f\Vert _L) \end{aligned}$$

at every sample point $\gamma _i$; this holds regardless of *f*. Suppose further the interpolation is successful (with smooth *f*, good points $\gamma _i$ and basis $\phi _i(z)$) in that $\Vert f-\hat{p}\Vert _\infty /\Vert f\Vert _\infty =O(u)$, where $\Vert f\Vert _\infty =\max _{z\in {\varOmega }}|f(z)|$ for a domain ${\varOmega }$. Then with a stable rootfinding algorithm for $\hat{p}$, one obtains stability in the computed roots: $p(\hat{r}_i) = O(u\Vert f\Vert _\infty )$. This shows $\hat{r}_i$ are the exact roots of a slightly perturbed input *f*. Rootfinding algorithms with proven stability include the companion [51] (for monomials) and colleague linearizations [32] (for Chebyshev).

For rational interpolation and polefinding, to our knowledge, stability in the context of polefinding and rational interpolation has been rarely discussed; [35], which connects the inaccuracies with the presence of ill-conditioned matrices, is one of the few, but their argument does not treat the backward stability of the rational interpolants $\frac{\hat{p}}{\hat{q}}$. Here we attempt to make a step forward and analyze backward stability for rational interpolation algorithms.

First we need to elucidate our goal. The presence of poles complicates the situation because, for example $\Vert f-\frac{\hat{p}}{\hat{q}}\Vert _\infty$ is infinity unless we compute the poles exactly, and this is true even for the linearized version $\Vert f\hat{q}-\hat{p}\Vert _\infty$. For this reason, sometimes rational interpolation is thought to be inherently ill-posed for a stable computation.

There is a natural workaround here: we allow for perturbation in both the numerator and denominator polynomials $\hat{p}$ and $\hat{q}$. We then analyze whether the rational interpolation is satisfied with small backward errors, that is,

5.2 $$\begin{aligned} f(\gamma _i) = \frac{\hat{p}(\gamma _i)+\Delta p(\gamma _i)}{\hat{q}(\gamma _i)+\Delta q(\gamma _i)},\quad \frac{\Vert \Delta p\Vert _L}{\Vert \hat{p}\Vert _L}=O(u),\ \frac{\Vert \Delta q\Vert _L}{\Vert \hat{q}\Vert _L}=O(u) \end{aligned}$$

for $i = 1,\ldots ,L,$. As before, we work with the linearized formulation.

### Definition 1

Let *f* be a meromorphic function. Given sample points $\{\gamma _i\}_{i=1}^L$ and computed polynomials $\hat{p}$, $\hat{q}$, we say that $\hat{p}/\hat{q}$ is a *stable rational interpolant* of *f* if there exist functions $\Delta q, \Delta p : \{\gamma _i\}_{i=1}^L\rightarrow \mathbb {C}$ such that

5.3 $$\begin{aligned} \begin{aligned}&f( \gamma _i)(\hat{q}( \gamma _i)+\Delta q( \gamma _i))-(\hat{p}(\gamma _i)+\Delta p(\gamma _i)) =0,\\&\frac{\Vert \Delta p\Vert _L}{\Vert \hat{p}\Vert _L} =O(u),\quad \frac{\Vert \Delta q\Vert _L}{\Vert \hat{q}\Vert _L} =O(u) . \end{aligned} \end{aligned}$$

We note that the requirement here is a rather weak condition: for example, it does not require that $\hat{p},\hat{q}$ are close to the correct *p*, *q* when $f=p/q$. Nonetheless, we shall see that many previous algorithms fail to satisfy them. We now give a necessary and sufficient condition for stability that is easy to work with.

### Lemma 1

5.4 $$\begin{aligned} |f(\gamma _i)\hat{q}(\gamma _i)-\hat{p}(\gamma _i)|=O(u)\max (|f(\gamma _i)|\Vert \hat{q}\Vert _L,\Vert \hat{p}\Vert _L) \end{aligned}$$

is a necessary and sufficient condition for $\hat{p}/\hat{q}$ to be a stable rational interpolant at $\gamma _i$ satisfying (5.3), for $i=1,\ldots ,L$.

(Proof) Suppose (5.4) is satisfied. Then, defining $\Delta p$ and $\Delta q$ by

5.5 $$\begin{aligned} ( \Delta p (\gamma _i ), \Delta q( \gamma _i ) ) = \left\{ \begin{array}{ll} (f(\gamma _i)\hat{q}(\gamma _i)-\hat{p}(\gamma _i) , \quad 0 ) &{} \text{ if } |f(\gamma _i)|\Vert \hat{q}\Vert _L\le \Vert \hat{p}\Vert _L\\ (0 , \quad - \frac{ f(\gamma _i ) \hat{q}( \gamma _i )- \hat{p}(\gamma _i) }{ f(\gamma _i ) } ) &{} \text{ if } |f(\gamma _i)| \Vert \hat{q}\Vert _L> \Vert \hat{p}\Vert _L, \end{array} \right. \end{aligned}$$

we obtain (5.3). Conversely, if $\Delta q$ and $\Delta p$ satisfy (5.3), then we have

5.6 $$\begin{aligned} |f(\gamma _i)\hat{q}(\gamma _i)-\hat{p}(\gamma _i)|= |f(\gamma _i) \Delta q(\gamma _i)- \Delta p(\gamma _i)|= O(u)\max (|f(\gamma _i)|\Vert \hat{q}\Vert _L,\Vert \hat{p}\Vert _L) . \end{aligned}$$

This proves the claim. $\square$

Below we analyze the stability of algorithms based on Lemma 1. In Sects. 5.1 and 5.2, to avoid the jarring complications due to the ill-conditioning of the Vandermonde matrix, we discuss the case where the sample points are the roots of unity and the polynomial basis is the monomials $\phi _i(z) = z^i$. Essentially the same argument carries over to other sets of sample points employed with an appropriate polynomial basis $\{\phi _i(z)\}$, such as Chebyshev-points sampling employing the Chebyshev polynomial basis.

### Instability of previous algorithms

Here we illustrate with the example of Chebfun’s ratinterp that previous algorithms can be numerically unstable, i.e., they do not necessarily satisfy (5.4) in Lemma 1. Recall that ratinterp computes $c_q$ in (2.11) as the null vector of $\widetilde{V}^*FV_{N+1}$.

Let us explain the numerical issue here. Let $\hat{c}_q$ be the computed null vector. Consider the Eq. (2.10) left-multiplied by the Vandermonde matrix *V*, which is unitary times $\sqrt{L}$. Taking into account the numerical errors, the equation can be written as

5.7 $$\begin{aligned} FV \begin{bmatrix} \hat{c}_q\\0 \end{bmatrix} = V \begin{bmatrix} \hat{c}_p\\\epsilon _{L-(m+1)} \end{bmatrix}, \end{aligned}$$

which we rewrite using $$\epsilon _L= V \big [ \begin{matrix} 0\\ \epsilon _{L-(m+1)} \end{matrix}\big ] $$ as

5.8 $$\begin{aligned} FV \begin{bmatrix} \hat{c}_q\\0 \end{bmatrix} - V \begin{bmatrix} \hat{c}_p\\0 \end{bmatrix} = \epsilon _L. \end{aligned}$$

The vectors $\epsilon _{L-(m+1)}$ and $\epsilon _L$ are zero when $\hat{c}_q$ is equal to the exact $c_q$, but $\epsilon _{L-(m+1)},\epsilon _L\ne 0$ due to numerical errors. Indeed, we see that the *i*th element of $\epsilon _L$ is $f(\gamma _i)\hat{q}(\gamma _i)-\hat{p}(\gamma _i)$, which is precisely the linearized interpolation residual in (5.4).

Now, the computed null vector $\hat{c}_q$ of the matrix $\widetilde{V}^*FV_{N+1}$ in (2.11) obtained by a stable algorithm such as the SVD generally satisfies the normwise condition

5.9 $$\begin{aligned} ( L\Vert \epsilon _{L}\Vert _2=)\ \Vert \widetilde{V}^*FV_{N+1}\hat{c}_q\Vert _2=O(u\Vert \widetilde{V}^*FV_{N+1}\Vert _2). \end{aligned}$$

Now since $\Vert V\Vert _2=\Vert V^*\Vert _2=\sqrt{L}=O(1)$, we have $\Vert \widetilde{V}^*FV_{N+1}\Vert _2= O(\max _i|f(\gamma _i)|)$. Thus $\Vert \epsilon _{L}\Vert _2=O(u\max _i|f(\gamma _i)|)$, which indicates that if $\max _i|f(\gamma _i)|\gg |f(\gamma _j)|$ for some *j*, then the interpolation residual for the *j*th equation is (for a constant $c_i=O(1)$)

$$\begin{aligned} f(\gamma _j)\hat{q}(\gamma _j)-\hat{p}(\gamma _j) =c_iu\max _i|f(\gamma _i)|\Vert \hat{q}\Vert _L\gg c_iu\max (|f(\gamma _j)|\Vert \hat{q}\Vert _L,\Vert \hat{p}\Vert _L), \end{aligned}$$

which violates the condition (5.4) for stability.

Although we do not present the details, such instability is present in most algorithms, including the *unscaled* naive method and RKFIT (with default weight $\tilde{D}$).

### Diagonal scaling and stability of ratfun and scaled naive method

Let us reconsider the eigenvalue problem (4.4) from a similar viewpoint, and we shall show that our approach of solving (4.4) employing diagonal scaling is immune to the instability just discussed, and ratfun gives a stable rational interpolation.

For simplicity we rewrite the eigenvalue problem (4.4) with diagonal scaling.^3^ $$ D\begin{bmatrix} A_1&A_2 \end{bmatrix}x =\lambda \begin{bmatrix} DB_1&O \end{bmatrix}x $$ as $DAx=\lambda DBx$. By the backward stability of the standard QZ algorithm, each computed eigenpair $(\hat{\xi }_i,\hat{x})$ satisfies

5.10 $$\begin{aligned} (DA+\Delta A)\hat{x}=\hat{\xi }_i (DB+\Delta B)\hat{x}, \quad \Vert \Delta A\Vert _2\le \epsilon \Vert DA\Vert _2, \Vert \Delta B\Vert _2\le \epsilon \Vert DB\Vert _2, \end{aligned}$$

in which $\epsilon$ denotes a constant of magnitude *O*(*u*).

To establish stability we need two preparations. First, we use an appropriate scaling of *f*. We can clearly scale $f\leftarrow \frac{1}{\kappa } f$ for any $\kappa >0$ without changing the poles and roots, and the analysis below will show that a good choice is one such that $\Vert c_p\Vert _2\approx \Vert c_q\Vert _2$. To be precise, it suffices to have

5.11 $$\begin{aligned} \frac{\Vert c_p\Vert _2}{\Vert c_q\Vert _2}=\Theta (1), \end{aligned}$$

which means $\frac{\Vert c_p\Vert _2}{\Vert c_q\Vert _2} =O(1)$ and $\frac{\Vert c_q\Vert _2}{\Vert c_p\Vert _2} =O(1).$ This means we expect $f=\Theta (1)$ holds at most of the sample points. In practice, we achieve (5.11) by sampling at sufficiently many points and taking $\kappa$ to be the median value $\text{ median }_L|f(\gamma )|$; this is adopted in the pseudocode of ratfun, Step 2 of Algorithm 4.1.

Second, as mentioned before, we choose the diagonal scaling matrix *D* as in (2.4), so that (since we scale *f* s.t. $\text{ median }_L|f(\gamma )|=1$) the *j*th diagonal $d_j$ is

5.12 $$\begin{aligned} d_j = \frac{1}{\max (|f(\gamma _i)|,1)}, \quad j = 1,\ldots , L. \end{aligned}$$

We are now ready to state our main stability result.

#### Theorem 1

Let *A*, *B* be as defined in (4.4) with $L=m+n+1$, where $\gamma _j=\exp (\frac{2\pi \mathrm {i}j}{L})$ and $\phi _i(z)=z^i$. Let $D=\text{ diag }(d_j)$ be as in (5.12), and let $(\hat{\xi }_k,\hat{x})$ with $|\hat{\xi }_k|=O(1)$ be a computed eigenpair such that (5.10) holds. Partition $$ \hat{x}=\big [ \begin{array}{c} \hat{c}_{\tilde{q}}\\ -\hat{c}_p \end{array} \big ]$$, where $\hat{c}_{\tilde{q}}\in \mathbb {C}^{n}$. Defining $\hat{p}=\sum _{j=0}^m\hat{c}_{p,j}z^j$, $\tilde{q}=\sum _{j=0}^{n-1}\hat{c}_{\tilde{q},j}z^j$ and $\hat{q}=(z-\hat{\xi }_k)\tilde{q}$ with coefficient vector $\hat{c}_q$, suppose that $\Vert \hat{c}_{q}\Vert _2/\Vert \hat{c}_{p}\Vert _2=\Theta (1)$. Then $\hat{p}/\hat{q}$ is a stable rational interpolant of *f*, that is, (5.4) is satisfied.

(Proof) By (5.10) we have

5.13 $$\begin{aligned} \Vert DA\hat{x}-\hat{\xi }_k DB\hat{x}\Vert _2=O(u(\Vert DA\Vert _2+\hat{\xi }_k\Vert DB\Vert _2)\Vert \hat{x}\Vert _2) =O(u\Vert \hat{x}\Vert _2), \end{aligned}$$

where we used the fact that $\Vert DA\Vert _2,\Vert DB\Vert _2$ and $|\xi _k|$ are all *O*(1). Now recalling (4.2), the *i*th element of $DA\hat{x}-\hat{\xi }_k DB\hat{x}$ is

$$\begin{aligned} (DA\hat{x}-\hat{\xi }_k DB\hat{x})_i&= d_i(\gamma _if(\gamma _i)\tilde{q}(\gamma _i) - \hat{p}(\gamma _i))- d_i \xi _kf(\gamma _i)\tilde{q}(\gamma _i)\\&=d_i(f(\gamma _i)(\gamma _i-\hat{\xi }_k)\tilde{q}(\gamma _i)-\hat{p}(\gamma _i))\\&=d_i(f(\gamma _i)\hat{q}(\gamma _i)-\hat{p}(\gamma _i)). \end{aligned}$$

This represents a *scaled* interpolation error, which is $O(u\Vert \hat{x}\Vert _2)$ by (5.13). Since $\gamma _i$ are roots of unity we have $\Vert \hat{p}\Vert _L= \sqrt{L}\Vert \hat{c}_{p}\Vert _2$ and $\Vert \hat{q}\Vert _L= \sqrt{L}\Vert \hat{c}_q\Vert _2$, so in view of Lemma 1, it suffices to show that

5.14 $$\begin{aligned} \frac{1}{d_i}\Vert \hat{x}\Vert _2=O(\max (|f(\gamma _i)|\Vert \hat{c}_q\Vert _2,\Vert \hat{c}_p\Vert _2)). \end{aligned}$$

Now since $$\Vert \hat{x}\Vert _2= \Vert \big [ \begin{array}{c} \hat{c}_{\tilde{q}}\\ \hat{c}_p \end{array} \big ] \Vert $$, using the assumption $\Vert \hat{c}_q\Vert _2/\Vert \hat{c}_p\Vert _2=\Theta (1)$ and the fact $\Vert \hat{c}_q\Vert _2/\Vert \hat{c}_{\tilde{q}}\Vert _2=\Theta (1)$, which follows from $|\hat{\xi }_k|=O(1)$, we have $\Vert \hat{x}\Vert _2/\Vert \hat{c}_p\Vert _2=O(1)$ and $\Vert \hat{x}\Vert /\Vert \hat{c}_q\Vert _L=O(1)$. Using these in Eq. (5.14) divided by $\Vert \hat{x}\Vert _2$, we see that it suffices to establish $\frac{1}{d_i}=O(\max (|f(\gamma _i)|,1))$, which indeed holds due to the choice of diagonal scaling (5.12).

Since the above argument is valid for every $i=1,\ldots ,L$, we conclude from Lemma 1 that $\hat{p}/\hat{q}$ is a stable rational interpolant of *f*. $\square$

We emphasize the crucial role that diagonal scaling plays in the stability analysis. We also note that the scaling such that $f\leftarrow cf$ is actually not necessary for the reduced eigenproblem (4.7) (without the diagonal scaling *D*), which is invariant under the scaling $f\leftarrow cf$.

*Stability of scaled naive method* The scaled naive method can also be proven to be stable. In this case the analysis is even simpler as the *j*th row of $$DC\big [ \begin{matrix} c_q\\ -c_p \end{matrix} \big ]$$, where *C* is as in (2.6), represents

5.15 $$\begin{aligned} d_j\left( \begin{bmatrix} FV_{n+1}&V_{m+1} \end{bmatrix} \begin{bmatrix} c_q\\ -c_p \end{bmatrix} \right) _j = d_j(f(\gamma _j)q(\gamma _j)-p(\gamma _j)). \end{aligned}$$

That is, the residual of each row is exactly the scaled interpolation error. Thus a null vector $$\big [ \begin{matrix} c_q\\ -c_p \end{matrix} \big ] $$ computed in a stable manner under the same assumptions as above [(5.10) and (5.11)] is automatically a stable rational interpolant.

However, for finding the poles, the additional process of finding the roots of *q* is necessary, and this can be a cause for further numerical instability. We discuss this further in Sect. 5.3.

*Barycentric formula* Finally, we mention the rational interpolation based on the barycentric formula [9–11, 41]

5.16 $$\begin{aligned} r(z)=\frac{\sum _{k=0}^n\frac{w_k}{z-\gamma _k}f_k }{\sum _{k=0}^n\frac{w_k}{z-\gamma _k}}, \end{aligned}$$

where $w=[w_1,\ldots ,w_n]$ are called the barycentric weights. For a general *w* (e.g. for randomly chosen *w*) the rational function *r*(*z*) in (5.16) is of type $(n,n)$. However, by choosing appropriate weights *w* one obtains an interpolant of desired type; Berrut and Mittelmann [10] show how such *w* can be found by a null vector of a matrix related to $$ \begin{bmatrix} FV_{n+1}&V_{m+1} \end{bmatrix}$$ as in (2.6). Antoulas and Anderson [1] introduce an algorithm for computing *w* to interpolate $r(\gamma _i)=f(\gamma _i)$, where $(\gamma _k)_{i=1}^n$ are taken to be half of the sample points and hence interpolation is achieved at 2*n* points. The recent AAA algorithm [33] chooses the points $(\gamma _k)_{i=1}^n$ in a greedy manner reduce the linearized error in the rational approximant.

As noted in [10], at the sample points $\gamma _k$ the barycentric formula (5.16) essentially gives an *exact* interpolation function, in that $r(\gamma _k)=f(\gamma _k)$ at all $\gamma _k$ (this holds regardless of the choice of *w* as long as $w_k\ne 0$). However, this is due to the representation of the rational function; finding the poles and obtaining *p*, *q* from (5.16) would induce further numerical errors. Below we focus our attention on algorithms that work with the coefficients of the rational interpolant.

### Accuracy of polefinder and effect of orthogonalization

For rational interpolation, we have described and identified two stable methods: ratfun and the scaled naive method.

Let us now turn to polefinding, and focus on the accuracy of the computed poles. ratfun finds the poles while simultaneously computing the rational approximant. By contrast, in the scaled naive method (or practically any other existing method for rational approximation) we first find the denominator polynomial *q*, then compute its roots. Intuitively this two-step approach should be more susceptible to numerical instability, and here we illustrate that this is indeed the case.

We compare two algorithms: scaled naive and ratfun. In ratfun, the QR factorization $DB_1=Q_{B_1}R_{B_1}$ in step 3 of Algorithm 4.1 is implicitly performing a change of basis for the polynomial *q*, so that discrete weighted orthogonality is achieved in the new basis. This has the possible disadvantage that the computed eigenvector in (4.15) contains the coefficients in the changed basis. However, crucially, for polefinding, this is not an issue at all, because the eigenvalues are unaffected. The change-of-basis is rather a benefit, because in the new basis the matrices $A_1,B_1$ are well-conditioned, which reduces numerical errors.

By contrast, this change-of-basis cannot be done for the naive method, because it requires the coefficients of *q* in a polynomial basis that is easy to work with for computing the roots.

*Numerical example* We illustrate the above discussion with an example. Let *f* be a rational function of the form (3.5) with $N=20$ poles in equispaced points on $[-1+\delta ,1-\delta ]$ with $\delta =10^{-3}$. We use the monomial basis but sample at Chebyshev points, whose number we vary. Therefore we are employing a “wrong” polynomial basis for the sample points; the “correct” one is the Chebyshev polynomials.

**Fig. 4:**
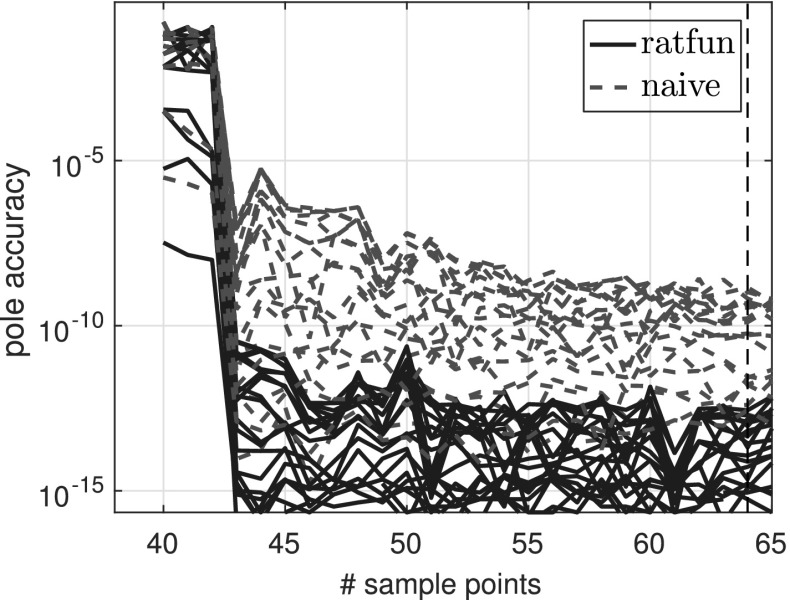
Error of 20 computed poles for ratfun and the naive method using the monomial basis. ratfun implicitly and automatically uses the appropriate polynomial basis to obtain accurate poles

Figure 4 shows the result with the two algorithms, showing the errors in the computed poles $\min _j|\xi _i-\hat{\xi }_j|$ for $i=1,\ldots , n$, as the number of sample points is varied. The diagonal scaling (5.12) is used for both algorithms; without this, the accuracy is significantly worse than in the figures.

ratfun clearly gives significantly more accurate results. The inaccuracy of the naive method is mainly due to the wrong choice of basis used to represent *q*. For example, by choosing the “correct” Chebyshev basis, the red plots become only slightly worse than ratfun. Indeed it is known that when looking for real roots of a polynomial, one is often advised to use Chebyshev polynomials instead of monomials.

The point here is that ratfun *automatically* finds an appropriate basis for the particular problem given: If a function *f* and a set of sample points are given, the QR factorization finds the appropriate basis. Indeed, if the QR factorization is not used in ratfun, the accuracy deteriorates significantly.

## Numerical experiments

We present further numerical experiments to illustrate the behavior of ratfun. We compare

1. ratfun: Algorithm 4.1,
2. The scaled naive method (2.8) with diagonal scaling (5.12), shown as naive,
3. Chebfun’s ratinterp command, shown as chebfun,
4. RKFIT [7, 8] with diagonal inputs $F=\text{ diag }(f(\gamma _i))$, $A= \text{ diag }(\gamma _i)$ and $v=[1,\ldots ,1]^\top$, with the default choice $\tilde{D}=I$ and maximum number of iterations set to 10 (increasing this made no noticeable difference).

All the experiments were conducted in MATLAB R2014a using IEEE double precision arithmetic with $u\approx 1.1\times 10^{-16}$, on a desktop machine with an Intel Core i7 Processor with four cores, and f16GB RAM.

For “easy” problems like those in Sect. 3.2, all the algorithms compute the poles and approximants reliably. Below we thus focus on more challenging problems.

**Fig. 5:**
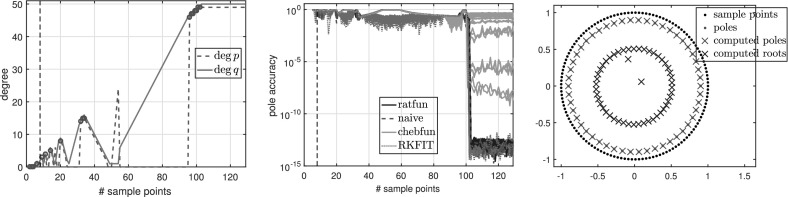
$f(z) = \sum _{i=1}^{N}\frac{1}{z-\xi _i}$, with 50 poles inside the unit disk. Left: numerical degrees of the rational approximants. Black dashed line indicates the number of sampled points $L$ in the degree determination process Algorithm 3.1; here $L=8$. ratfun(f) returns the incorrect type (0, 2); see comment in the text. Middle: error of computed poles $\min _j|\xi _i-\hat{\xi }_j|$ with ratfun(f,gam) sampling 128 points. Right: sample points, poles and roots

*High-degree examples* We consider a moderately high-degree example, where we take *f* as in (3.5) with $N=50$. The results are in Fig. 5.

With a sufficient number of sample points $L\ge 103$, ratfun finds the type of the rational approximant and computes the poles and roots stably. Here and below, the roots are computed simply by finding the poles of 1 / *f* by ratfun; the other processes described in Sect. 4.6 had similar performances.

It is worth observing that most computed roots turned out to lie on a circle of radius about 0.5. This may look bizarre at first sight, as one easily sees that the only zero of *f* is at $z=0$. This can be explained by eigenvalue perturbation theory: the zero of *f* has multiplicity $N-1=49$, so the eigenvalue problem for computing it attempts to find the eigenvalue of algebraic multiplicity 49. Now it is well known [43, Ch. 5] that the eigenvalues of algebraic multiplicity *k* and geometric multiplicity 1, which are essentially eigenvalues of a $k\times k$ Jordan block, can be perturbed by $O(\epsilon ^{1/k})$ by a perturbation in the matrix of norm $O(\epsilon )$. The QZ algorithm computed the roots in a backward stable manner, but the small perturbation is enough to perturb them by $u^{\frac{1}{49}}\approx 0.4725$. The reason the roots appear to lie systematically on a circle is that the eigenvalues of a Jordan block are extremely sensitive to perturbation in the bottom-left element, but much less so in other positions.

Note in the left figure that with insufficient sample points $L< 100$ the type finder outputs an incorrect output. In view of Lemma 4, this happens when the number of sample points *L* was less than the necessary $\max \{ M+ n , m + N\} + 1$, but the function *f* and sample points $\gamma _i$ happened to (e.g. by symmetry) make the matrix *C* rank-deficient, and so at $\gamma _i$ the function behaved as if it is a lower-type rational function. The same was observed in Fig. 1, and the problem is pronounced here. Indeed the degree determination Algorithm 3.1 indicated a numerical degree of (0, 2) after sampling initially at the eigth roots of unity. We have not overcome this issue completely; indeed such difficulty is present even in the polynomial case [48]; for example when a highly oscillatory function *f* happened to be 0 at all the initial sample points. Perhaps some further insurance policy is needed to ensure that the type obtained by Algorithm 3.1 is appropriate, such as sampling *f* at a few more random points [2]. One could also try typefind(f,tol,L) for neighboring values of *L* and accept only if they are the same. These remedies, while effective, cannot be proven to be fool-proof.

**Fig. 6:**
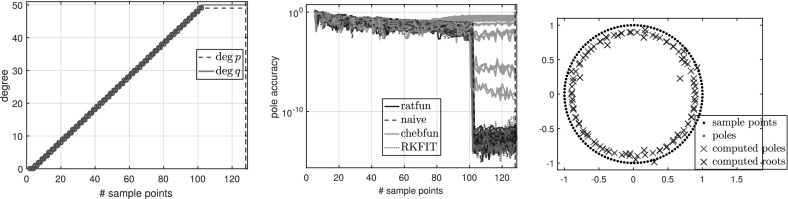
$f(z) = \sum _{i=1}^{N}\frac{r_i}{z-\xi _i}$ with random residues $r_i$. Left: numerical degrees of the rational approximants. ratfun(f) samples at $2^7$ points and returns the correct type (49, 50). Middle: error of computed poles $\min _j|\xi _i-\hat{\xi }_j|$. Right: sample points, poles and roots

Nonetheless, this is a rather contrived example with high symmetry, which generically would not happen. For example, if we take the residues of each term in (3.6) to be random numbers, we obtain Fig. 6, for which an appropriate type is chosen. In both cases, once a sufficient number of sampled points is taken, ratfun finds the correct poles and rational intepolant.

*When* *f* *has poles far away from sample points* Another possible difficulty is when *f* has a pole far away from the sample points.

**Fig. 7:**
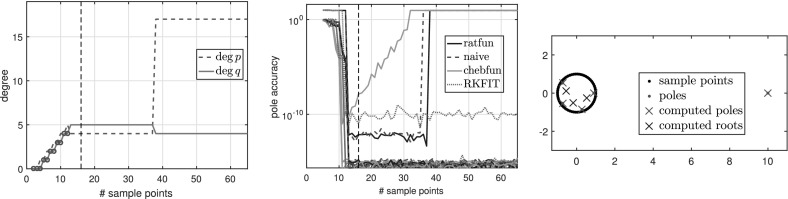
*f* with a pole far outside the unit disk. Left: numerical degrees of the rational approximants. ratfun(f) samples at $2^4$ points and returns the type (4, 5). Middle: error of computed poles $\min _j|\xi _i-\hat{\xi }_j|$. The pole that eventually gets lost by ratfun and naive corresponds to $\xi _1=10$, the pole far from the sample points. Right: sample points, poles and roots

To examine the behavior of our algorithm in such cases we take *f* as in (3.5) of type (4, 5), but we now set one pole to be far by taking $\xi _1=10$. Figure 7 shows the results.

Again, with a sufficient number of sample points we obtain a rational approximant of correct type (4, 5). In the middle error plot in Fig. 7, the poles inside the unit disk are computed accurately to machine precision. By contrast, the pole $\xi _1=10$ is computed with poorer accuracy. Loss of accuracy for poles outside the unit disk is a typical phenomenon, and the accuracy worsens rapidly if we take $|\xi _1|$ larger or let *f* be of higher type. This observation can be explained via eigenvalue conditioning analysis, which shows the condition numbers of the eigenvalues of (4.4) corresponding to poles outside the unit disk grow exponentially with base $|\xi _i|$ and exponent $m+n$, whereas those of eigenvalues inside the unit circle decrease (slowly) with $m+n$. The analysis is presented in “Appendix C”.

Recall that ratfun finds a numerical type by Algortihm 3.1. As explained in Sect. 3.1, there can be other numerical types for *f* that may be appropriate: Indeed, if we sample at many more points than necessary (i.e., typefind(f,tol,L) with $L\gg m+n+1$), ratfun eventually ignores the outlying pole and converges to a rational function of type $(m,4)$ where $m$ is large. That is, the computed outcome has lower denominator degree than that of the exact type 5; recall the experiment with (3.7) with a similar discussion. This can be explained as follows. By a standard result in complex analysis [34, Ch. 9], inside the unit disk a meromorphic function *f* can be written as

6.1 $$\begin{aligned} f(z) = \sum _{|\xi _i|\le 1}\sum _{j}\frac{a_{i,j}}{(z-\xi _i)^j}+ p_f(z). \end{aligned}$$

The sum is taken over the poles inside the unit disk. Here $p_f(z)$ is a power series, obtained e.g. as a Taylor series of $f(z) - \sum _{|\xi _i|\le 1}\sum _{j}\frac{a_{i,j}}{(z-\xi _i)^j}$, which converges inside a disk centered at the origin and of radius $|\xi _0|$, where $\xi _0$ is the pole closest to the origin besides those with $|\xi _i|\le 1$; here $|\xi _0|=10$. Therefore, near the sample points (the unit circle), *f* behaves as if it is a sum of terms $1/(z-\xi _i)$ with $|\xi _i|\le 1$, and an analytic function.

From a practical viewpoint, this example suggests that we should locate the sample points near the poles of interest. For example, we can find the pole $\xi _1=10$ accurately in the above example by taking the sample points to lie on a circle centered around 10.

*When a sample point is near a pole* This example illustrates how existing algorithms lose accuracy when a sample point is near a pole.

We form a rational function $f(z)=\frac{\prod _{i=0}^M(z-r_i)}{\prod _{i=0}^N(z-\xi _i)}$ where the roots $r_i$ and poles $\xi _i$ are generated randomly to lie in the unit disk. Here we take $M=4, N=5$, and let the sample points be equispaced points on the unit circle. We then reset one pole to be $1+10^{-13}$, forcing it to lie close to a sample point.

ratfun and the naive method compute the poles much more accurately than the other methods. This is largely due to the diagonal scaling discussed in Sect. 5; indeed, if we turn off the diagonal scaling and take $D=I$, the accuracy deteriorates for both ratfun and the naive methods to about the same as Chebfun’s ratinterp and RKFIT.

**Fig. 8:**
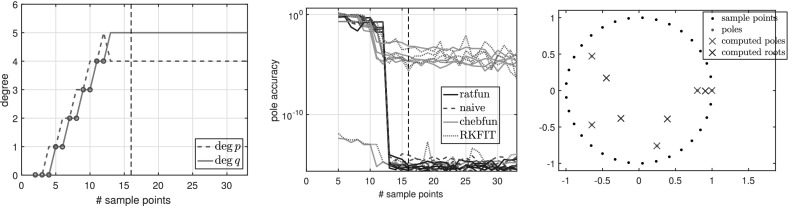
*f* with a pole close to a sample point. ratfun(f) samples at $2^4$ points and returns the correct type. Left: numerical degrees of the rational approximants. Middle: error of computed poles $\min _j|\xi _i-\hat{\xi }_j|$. Right: sample points, poles and roots

We believe that with RKFIT, which allows for tuning various inputs and parameters, it is possible to obtain accurate results if appropriate parameters are provided, such as $\tilde{D}$; recall the discussion in Sect. 2.3. The point here is that our analysis revealed an appropriate choice (Fig. 8).

*Rational functions with poles of order* $>1$ When *f* is memorophic but has poles $\xi _i$ of order $d_i>1$, the generalized eigenvalue problems (4.4) and (4.15) have an eigenvalue $\xi _i$ of the same multiplicity $d_i$. Here we examine the behavior of our algorithm in such cases.

We generate the function *f* simply by squaring the function in (3.5), that is, $f(z) = ( \sum _{j=1}^{5 }\frac{1}{z-\xi _j})^2$ with $\xi _j=0.9\exp (2\pi \mathrm {i} j/5 )$. Then *f* has 5 poles, all of which are of order 2.

**Fig. 9:**
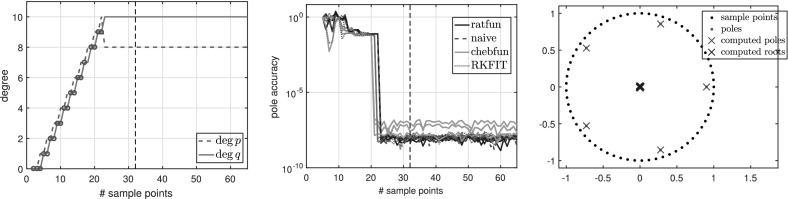
*f* with double poles. Left: numerical degrees of the rational approximants. Middle: error of computed poles $\min _j|\xi _i-\hat{\xi }_j|$. Right: sample points, poles and roots

Observe that all the algorithms, including ratfun, find the poles with accuracy $O(\sqrt{\epsilon })$, which is what one would expect from a backward stable algorithm: the poles $\xi _i$ of order 2 result in an eigenvalue with Jordan block of size 2, and perturbation of $\epsilon$ in the matrices perturb such eigenvalues by $O(\sqrt{\epsilon })$ (Fig. 9).

*Non-meromorphic functions* Although we have started our discussion assuming *f* is a meromorphic function in the unit disk, our algorithm can be applied to *f* with singularities other than poles, as long as *f* can be evaluated at the sample points. We now explore such cases by examining functions with a branch cut, or an essential singularity.

First let *f* have a log-type branch cut

**Fig. 10:**
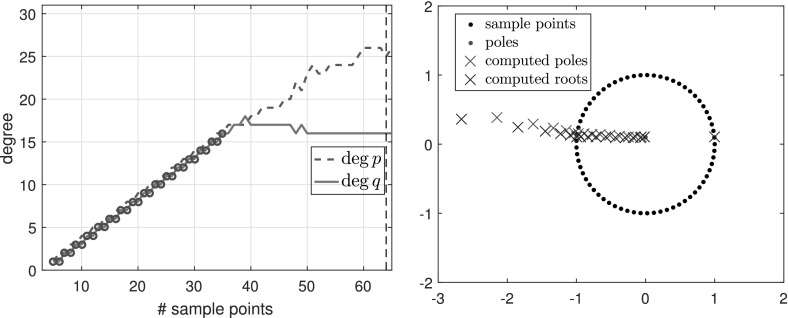
*f* with a log-type branch cut. ratfun(f) samples at $2^5$ points and determines the numerical type (14, 14). Left: numerical degrees of the rational approximants. Right: sample points, poles and roots

6.2 $$\begin{aligned} f(z) = \log \left( z-\frac{1}{10}i\right) , \end{aligned}$$

which has a branch cut on $z=\frac{1}{10}i-\mathbb {R}_+$. Figure 10 shows the results. Observe that spurious poles and roots appear along the branch cut; we suspect this is related to a similar phenomenon known for Padé approximants of functions with branch cuts [42].

For a function with an essential singularity, we examine the standard example

6.3 $$\begin{aligned} f(z) = \exp \left( \frac{1}{z}\right) . \end{aligned}$$

**Fig. 11:**
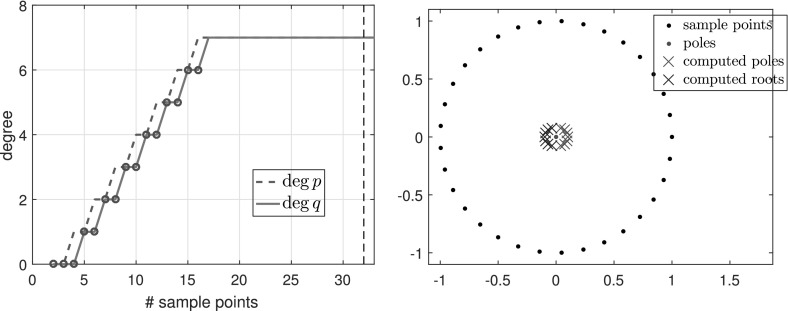
*f* with an essential singularity. Left: numerical degrees of the rational approximants. ratfun(f) samples at $2^4$ points and determines the numerical type (7, 7). Right: sample points, poles and roots

The results are in Fig. 11. Again, spurious poles and roots appear near the singularity point 0, but away from the singularity *f* is bounded and analytic, and is well approximated by the rational interpolant. This is no surprise as $\exp (1/z)$ behaves as a completely analytic function on the unit circle.

**Fig. 12:**
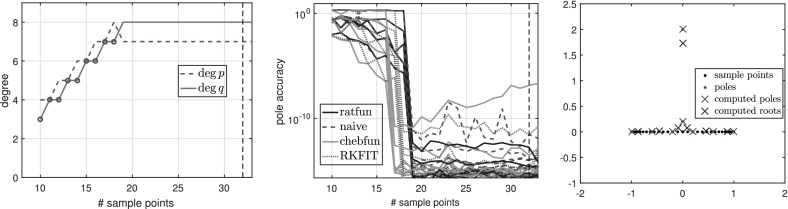
Sampled at Chebyshev points. ratfun(f) samples at $2^5$ points and determines the correct type (8, 7). The pole at $2\mathrm {i}$ loses accuracy as we sample more and increase $m$

*Sample points at Chebyshev points* In this example the sample points are taken to be the Chebyshev points $\gamma _j = \cos (\frac{\pi (j-1)}{L-1})$ and the polynomial basis is Chebyshev polynomials $\phi _i(x)=T_i(x)$. This is numerically recommended when most poles lie on the real axis. In this example *f* is again as in (3.5), with 6 equispaced poles on $[-1+10^{-2},1-10^{-2}]$, along with complex poles at $0.2\mathrm {i}$ and $2\mathrm {i}$. The results are in Fig. 12. For such functions, sampling at Chebyshev points give better accuracy than roots of unity.

Although not shown in the figure, the accuracy of poles far from $[-1,1]$ worsens rapidly as the poles lie farther away, or the function type increases. This is analogous to the observation made in Fig. 7: the poles far from the sample points will eventually get ignored (here the poles that converge are those within a narrow ellipse that covers the real interval $[-1,1]$).

**Fig. 13:**
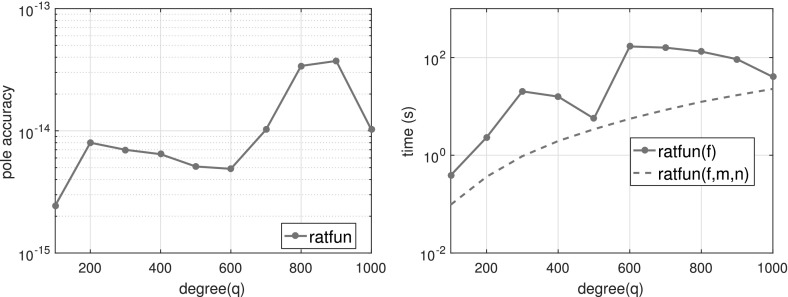
High-degree example, accuracy of computed poles $\max _j|\xi _j-\hat{\xi }_j|$ (left) and runtime (right)

*Speed illustration* Here we examine the speed and accuracy of ratfun for high-degree rational functions. We take *f* to be as in (3.5) with poles $\xi$ being the Chebyshev points, scaled by $1-10^{-5}$, and vary the number of points (i.e., the degree of *q*) from 100 to 1000. We sample at the Chebyshev points. In order to examine the breakdown of the runtime we present the runtime for (1) ratfun(f), which inputs only the function (hence starts by finding the type), and (2) ratfun(f,m,n), which inputs the correct type (and hence bypasses the type determination). The results are in Fig. 13. This example illustrates that ratfun can work with rational functions of quite high degree, and that the degree determination step often takes up a dominant part of the runtime.

*Eigenvalues of a matrix via the resolvent* One use of rational approximation and polefinding that has been attracting recent interest [4] is in finding eigenvalues of a matrix *A* or matrix pencil $A-\lambda B$ via finding the poles of the projected resolvent $u^\top (A-zI)^{-1}v$ or $u^\top (A-zB)^{-1}v$, where *u*, *v* are some vectors (usually random). We have applied our algorithm to this problem, and observed that it works. However, usually it is not superior to the algorithm presented in [4], which combines a rational filter function with a block subspace whose dimension is proportional to the estimated number of eigenvalues in the region of interest. The distinct feature in [4] (and also the FEAST eigensolver [36]) is that the algorithm works with the subspaces $(A-zB)^{-1}V$ instead of the function $u^\top (A-zB)^{-1}v$, and this is crucial to overcome the difficulty associated with a nearly multiple eigenvalue. We suspect that an extension of our algorithm to work with block subspaces would be possible; we leave this for future work.

## The Kronecker canonical form

Here we analyze in detail the generalized eigenvalue problem (4.4) and derive its Kronecker canonical form [17, Ch. 4.5.2]. It shows in particular that multiple poles (if any) can be computed along with their multiplicities, at least in exact arithmetic.

Here, let *f* be a rational function $f(z) = \frac{p(z)}{q(z)} \in \mathcal {R}_{m,n}$, where *p*(*z*) and *q*(*z*) have no common divisors except for constants (i.e., $f=p/q$ is an *irreducible* expression). Then *p*(*z*) and *q*(*z*) are in the following form:

A.1 $$\begin{aligned} p(z)= \alpha \prod _{j=1}^J ( z - \eta _j )^{c_j}, \quad q(z) = \beta \prod _{k=1}^K ( z - \xi _k )^{d_k}, \end{aligned}$$

where $\eta _1 , \ldots , \eta _J , \xi _1, \ldots , \xi _K \in \mathbb {C}$ are distinct and $\alpha , \beta \in \mathbb {C} \setminus \{ 0 \}$. Each $\eta _j$ is a root of *f* of multiplicity $c_j$ and each $\xi _k$ is a pole of order $d_k$. Note that $M= \deg p = \sum _{j=1}^J c_j \le m$ and $N= \deg q = \sum _{k=1} ^ K d_k \le n$ since $f \in \mathcal {R}_{m,n}$. For simplicity we analyze the case where $L=m+n+1$.

### Proposition 2

The matrix pencil $A - \lambda B = [A_1, A_2] - \lambda [ B_1 , O ]$ is strictly equivalent to the matrix pencil where

A.3 $$\begin{aligned} H_k(\lambda )&= \left. \left[ \begin{array}{cccc} \xi _k - \lambda &{} 1 &{} &{} \\ &{} \xi _k - \lambda &{} \ddots &{} \\ &{} &{} \ddots &{} 1 \\ &{} &{} &{} \xi _k - \lambda \end{array} \right] \right. \in \mathbb {C}[\lambda ]^{d_k \times d_k}, \quad ( k=1, \ldots ,K )&\end{aligned}$$

A.4 $$\begin{aligned} U(\lambda )&= \left. \left[ \begin{array}{cccc} -\lambda &{} &{} &{} \\ 1 &{} -\lambda &{} &{} \\ &{} 1 &{} \ddots &{} \\ &{} &{} \ddots &{} -\lambda \\ &{} &{} &{} 1 \end{array} \right] \right. \in \mathbb {C}[\lambda ]^{(n- N+ 1) \times (n- N)}. \end{aligned}$$

(Proof) It suffices to show that there exist nonsingular constant matrices $P, Q \in \mathbb {C}^{L\times L}$ satisfying $(A - \lambda B ) P = Q F( \lambda )$. We will construct such *P* and *Q* in two steps: (1) construct $P_k , Q_k \in \mathbb {C}^{(m+n+1) \times d_k }$ satisfying

A.5 $$\begin{aligned} (A - \lambda B ) P_k = Q_k H_k ( \lambda ) \end{aligned}$$

for $k=1, \ldots , K$, and (2) construct $S \in \mathbb {C}^{L\times (L-N)}$, $T_1 \in \mathbb {C}^{L\times (m+1)}$ and $T_2 \in \mathbb {C}^{L\times (n-N)}$ such that (using MATLAB notation)

A.6 $$\begin{aligned} (A - \lambda B) T_1&= S(:,1:m+1),&\end{aligned}$$

A.7 $$\begin{aligned} (A- \lambda B) T_2&= S(:, M+1: n+M-N) U(\lambda ) .&\end{aligned}$$

Then, *P* and *Q* defined by

A.8 $$\begin{aligned} P=[P_1, \ldots , P_K , T_1, T_2], \quad Q=[ Q_1, \ldots , Q_K , S ] \end{aligned}$$

satisfy $$(A - \lambda B ) P = Q F( \lambda ) $$ .

Before discussing Step (1), let us introduce some notation. For functions *g*(*z*) in *z* and sample points $\{\gamma _i\}_{i=1}^{L}$, define the “vector of values” as

A.9 $$\begin{aligned} v(g(z)):= [g(\gamma _1), \ldots , g( \gamma _{L} ) ] ^{\top } \in \mathbb {C}^{L} . \end{aligned}$$

Similarly, for $p_1(z) = \sum _{j=0}^{n-1} a_j z^j \in \mathcal {P}_{n-1}$ and $p_2 (z) = \sum _{j=0}^{m} b_j z^j \in \mathcal {P}_{m}$, define the “vector of coefficients” as

A.10 $$\begin{aligned} c(p_1, p_2):= [ a_0, \ldots ,a_{n-1}, b_0 , \ldots , b_m] ^{\top } \in \mathbb {C}^{L} . \end{aligned}$$

Then, it holds for arbitrary $\lambda$ that

A.11 $$\begin{aligned} (A - \lambda B)c (p_1, p_2 ) = v ( (z - \lambda ) f(z) p_1(z) + p_2(z) ). \end{aligned}$$

(1) We will construct $P_k , Q_k \in \mathbb {C}^{(L) \times d_k }$ satisfying (A.5) for each $k \in \{ 1, \ldots , K \}$. For $d=0,1, \ldots , d_k$, define polynomials $q_k ^{(d)} \in \mathcal {P}_{n-1}$ by

A.12 $$\begin{aligned} q_k ^{(d)} (z) := q(z) \sum _{j=1}^d \frac{1}{ ( z-\xi _k )^j }, \end{aligned}$$

where we define $q_k ^{(0)}=0$. Then, for $d=1, \ldots , d_k$, the polynomial $q_k ^{(d)}$ satisfies

$$\begin{aligned} (z - \lambda ) f(z) q_k ^{(d)} (z) - p(z)&= p(z) \left( \sum _{j=1}^d \frac{z-\lambda }{( z-\xi _k )^j} - 1 \right) \\&= p(z) \left( \sum _{j=1}^d \frac{(z- \xi _k ) + (\xi _k - \lambda )}{( z-\xi _k )^j} - 1 \right) \\&= p(z) \left( (\xi _k - \lambda ) \sum _{j=1}^d \frac{1}{( z-\xi _k )^j} + \sum _{j=1}^d \frac{1}{( z-\xi _k )^{j-1}} - 1 \right) \\&= p(z) \left( (\xi _k - \lambda ) \sum _{j=1}^d \frac{1}{( z-\xi _k )^j} + \sum _{j=1}^{d-1} \frac{1}{( z-\xi _k )^{j}} \right) \\&= (\xi _k - \lambda ) f(z) q_k^{(d)}(z) + f(z) q_k^{(d-1)}(z) . \end{aligned}$$

From this equation and (A.11), we see that $P_k, Q_k\in \mathbb {C}^{(L)\times d_k}$ defined by

A.13 $$\begin{aligned} P_k&= [ c( q_k ^{(1)}(z ), - p(z) ) , \ldots , c( q_k ^{(d_k)}(z ), - p(z) ) ], \nonumber \\ Q_k&= [ v( f(z) q_k ^{(1)}(z ) ) , \ldots , v( f(z) q_k ^{(d_k)}(z ) ) ] \end{aligned}$$

satisfy

$$\begin{aligned} (A - \lambda B) P_k( :, 1 )&= ( \zeta _k - \lambda ) Q_k ( :, 1 ),\\ (A - \lambda B) P_k( :, j )&= ( \zeta _k - \lambda ) Q_k ( :, j ) + Q_k(:, j-1), \quad j=2, \ldots , d_k , \end{aligned}$$

which means that $(A - \lambda B) P_k = Q_k H_k(\lambda )$.

(2) Define $S \in \mathbb {C}^{L\times (L-N)}$, $T_1 \in \mathbb {C}^{L\times (m+1)}$ and $T_2 \in \mathbb {C}^{L\times (n-N)}$ by

A.14 $$\begin{aligned} T_1&= [ c( 0, z^0 ), \ldots , c(0, z^{M- 1} ), c( 0, z^0 p(z) ), \ldots , c( 0, z^{m-M} p(z) ) ] , \end{aligned}$$

A.15 $$\begin{aligned} T_2&= [ c(z^0 q(z) , 0 ), \ldots , c(z^{n-N-1 } q(z), 0) ], \end{aligned}$$

A.16 $$\begin{aligned} S&= [ v(z^0) , \ldots , v( z^{M- 1} ), v( z^0 p(z) ), \ldots , v( z^{m+n-N-M} p(z) ) ]. \end{aligned}$$

By substituting $p_1=0$ into (A.11), we obtain $(A - \lambda B) c( 0, p_2(z)) = v( p_2(z) )$ for an arbitrary $p_2 \in \mathcal {P}_{m}$, which implies (A.6). Since (A.11) gives

A.17 $$\begin{aligned} (A - \lambda B)c (z^{j-1} q(z), 0) = v((z-\lambda )f(z) z^{j-1} q(z)) = - \lambda v (z^{j-1} p(z))+ v (z^{j}p(z)) \end{aligned}$$

for $j=1, \ldots , n-N$, we have (A.7).

From (A.5), (A.6) and (A.7), *P* and *Q* defined by (A.8) satisfy $(A- \lambda B)P=QF(\lambda )$.

It remains to show that *P* and *Q* are nonsingular. This is proven in the next lemma. $\square$

### Lemma 2

The matrices *P* and *Q* defined by (A.13), (A.16), (A.14), (A.15) and (A.8) are nonsingular.

(Proof) Since $T_1 (1:n,:)$ is a zero matrix and $T_1 (n+1: m+n+1, :)$ is an upper triangular matrix with non-zero diagonal entries, $P= [ P_1 , \ldots , P_K , T_1, T_2 ]$ is nonsingular if and only if $W= [P_1, \ldots , P_K, T_2](1:n,:)$ is nonsingular. We shall prove that *R* is nonsingular by showing that $W x = 0$ implies $x = 0$. Write $x = [x_1^{(1)}, \ldots ,x_1^{(d_1)}, x_2^{(1)}, \ldots , x_K^{(d_K)}, y_1, \ldots , y_{n-N} ]^\top \in \mathbb {C}^{n}$. If $W x = 0$, from the definitions (A.13), (A.15) of $P_k$ and $T_2$, all the coefficients of the polynomial

A.18 $$\begin{aligned} p_x (z) = \sum _{k=1}^K \sum _{j=1}^{d_k} x_k ^{(j)} q_k^{(j)} (z) + \sum _{i=1}^{n-N} y_i z^{i-1}q(z) \end{aligned}$$

are equal to zero, and hence $p_x$ is the zero polynomial. Therefore, the rational function

A.19 $$\begin{aligned} \frac{p_x (z)}{q(z)} {=} \sum _{k=1}^K \sum _{j=1}^{d_k} x_k ^{(j)} \sum _{s=1}^j \frac{1}{(z - \xi _k)^s} + \sum _{i=1}^{n-N} y_i z^{i-1} = \sum _{k=1}^K \sum _{s=1}^{d_k} \frac{ \sum _{j=s}^{d_k} x_{k}^{(j)} }{(z - \xi _k)^s} + \sum _{i=1}^{n-N} y_i z^{i-1} \end{aligned}$$

is also the zero function. This means that

A.20 $$\begin{aligned} \sum _{j=s}^{d_k} x_{k}^{(j)}&= 0 \quad ( k=1, \ldots , K, s=1, \ldots , d_k ) , \end{aligned}$$

A.21 $$\begin{aligned} y_i&= 0 \quad ( i= 1, \ldots , n- N) , \end{aligned}$$

which implies that all the elements in *x* are zero. It follows from the above argument that *P* is nonsingular.

We prove that *Q* is nonsingular similarly by showing that $Qx = 0$ implies $x=0$. Write $x = [x_1^{(1)}, \ldots ,x_1^{(d_1)}, x_2^{(1)}, \ldots , x_K^{(d_K)}, y_1, \ldots , y_{M}, w_1, \ldots , w_{m+n- M-N+1} ]^\top \in \mathbb {C}^{m+n+1}$. If $Q x = 0$, from the definitions (A.13) and (A.16) of $Q_k$ and *S*, we have

A.22 $$\begin{aligned} g_x ( z ) := \sum _{k=1}^K \sum _{j=1}^{d_k} x_k ^{(j)} f(z) q_k^{(j)} (z) + \sum _{i=1}^{M} y_i z^{i-1} + \sum _{l=1}^{m+n-M-N+1 } w_l z^{l-1} p(z) = 0 \end{aligned}$$

for $z = \gamma _1 , \ldots , \gamma _{m+n+1}$. By multiplying *q*(*z*) to both sides, we have

A.23 $$\begin{aligned} h_x ( z)&:= q(z)g_x ( z ) = \sum _{k=1}^K \sum _{j=1}^{d_k} p(z) x_k ^{(j)} q_k^{(j)} (z) + \sum _{i=1}^{M} y_i z^{i-1}q(z) \nonumber \\&\quad +\, \sum _{l=1}^{m+n-M-N+1 } w_l z^{l-1} q(z) p(z) = 0 \end{aligned}$$

for $z = \gamma _1 , \ldots , \gamma _{m+n+1}$. Since $h_x$ is a polynomial of degree at most $m+n$ and take on the value 0 at $m+n+1$ distinct points, $h_x$ must be the zero polynomial. Using this fact we obtain $x=0$ as in the case of *P*, and hence *Q* is nonsingular. $\square$

### Corollary 1

The Kronecker canonical form of the pencil $A - \lambda B$ is as follows:

A.24

where $$s_1 = M+ \max \{ 0 , m-n- M+ N\}$$, $$s_2 = \max \{ 0, -m+n+ M-N\}+1 $$, $$s_3 = \min \{ m-M, n-N\}$$,

A.25 $$\begin{aligned} J _{s_2} (\lambda )&= \left. \left[ \begin{array}{cccc} 1 &{} - \lambda &{} &{} \\ &{} 1 &{} \ddots &{} \\ &{} &{} \ddots &{} - \lambda \\ &{} &{} &{} 1 \end{array} \right] \right\} s_2 ,&\end{aligned}$$

A.26 $$\begin{aligned} L_j (\lambda )&= [ 1 , - \lambda ], \quad j=1, \ldots , s_3&\end{aligned}$$

and $H_j (\lambda )$ is defined by (A.3) .

(Proof) We can transform the $(m+n-N) \times (m+n-N)$ lower-right block of $L( \lambda )$ to obtain the Kronecker canonical form as follows:

$$\begin{aligned} \begin{bmatrix} 1&&&&\\&1&&-\lambda&&\\&1&1&-\lambda&\\&&1&1&-\lambda&\\&&&1&-\lambda \\&&&&1 \\&&&&\\&&&&\\&&&&\end{bmatrix} \begin{array}{c} \longrightarrow \\ \text {erase } s_3 \text { 1's} \end{array} \begin{bmatrix} 1&&&&\\&1&&-\lambda&&\\&1&&-\lambda&\\&&1&&-\lambda&\\&&&1&-\lambda \\&&&&1 \\&&&&\\&&&&\\&&&&\end{bmatrix} \\ \begin{array}{c} \longrightarrow \\ \text {column} \\ \text {permutation} \end{array} \begin{bmatrix} 1&&&&\\&&1&-\lambda&\\&&&1&-\lambda \\&1&-\lambda&&&\\&1&-\lambda&&\\&&1&&\\&&&&\\&&&&\\&&&&\end{bmatrix} \begin{array}{c} \longrightarrow \\ \text {row} \\ \text {permutation} \end{array} \begin{bmatrix} 1&&&&\\&1&-\lambda&&&\\&1&-\lambda&&\\&&1&&\\&&1&-\lambda&\\&&&1&-\lambda \\&&&&\\&&&&\\&&&&\end{bmatrix} . \end{aligned}$$

## Rank of the rational interpolation matrix

Here we analyze the rank of the matrix in (2.6) and derive (3.2). For a rational function $f=r$ sampled at $\{ \gamma _1 , \ldots , \gamma _L\}$, define $C_{mn}(r) \in \mathbb {C}^{L\times (m+n+2)}$ by $C_{mn}(r) = [ F V_{n+1} , V_{m+1} ]$, where $F = \mathrm {diag} ( r(\gamma _1 ), \ldots ,r( \gamma _{L}) )$. Then we have

B.1 $$\begin{aligned} \mathrm {range} ( C_{mn}(r) ) = \{ v(r p_1 + p_2 ) \mid p_1 \in \mathcal {P}_n , p_2 \in \mathcal {P}_m \}, \end{aligned}$$

where we define $v(h) = [ h( \gamma _1 ), \ldots , h(\gamma _L) ]^{\top } \in \mathbb {C}^L$ for functions *h*.

In this section we focus on the case where *r* is a rational function, and assume that *r* does not have poles coinciding with the sample points $\gamma _1 , \ldots , \gamma _L$. When $r=p/q$ is an irreducible expression, the degrees $M, N$ of *p*, *q* are uniquely determined, dependening only on *r*. For these $M$ and $N$, we say that *r* is of *exact type* $(M, N)$.

Below we summarize the properties of the matrix $C_{mn}(r)$ when *r* is a rational function of exact type $(M, N)$ and has an irreducible expression $r=p/q$. Note that *r* is not necessarily in $\mathcal {R}_{mn}$.

From (B.1), we have

B.2 $$\begin{aligned} \mathrm {range} ( C_{mn}(r)) = \{ v( ( p p_1 + q p_2 )/ q ) \mid p_1 \in \mathcal {P}_n , p_2 \in \mathcal {P}_m \}. \end{aligned}$$

Let $D:=\max \{ M+ n , m + N\}$. Defining a subspace $\mathcal {Q}$ of $\mathcal {P}_D$ by

B.3 $$\begin{aligned} \mathcal {Q}:=\{ p p_1 + q p_2 \mid p_1 \in \mathcal {P}_n , p_2 \in \mathcal {P}_m \}, \end{aligned}$$

we obtain

B.4 $$\begin{aligned} \mathrm {range} ( C_{mn}(r)) = \{ v( p_3 / q ) \mid p_3 \in \mathcal {Q} \}. \end{aligned}$$

### Lemma 3

If $r \in \mathcal {R}_{mn}$, i.e., $M\le m$ and $N\le n$, then $\mathcal {Q} = \mathcal {P}_D$.

(Proof) It suffices to show that $\dim \mathcal {Q} \ge D+1$. Since $\mathcal {Q} = p \mathcal {P}_n + q \mathcal {P}_m$, we have

B.5 $$\begin{aligned} \dim ( \mathcal {Q} )&= \dim ( p \mathcal {P}_n ) + \dim ( q \mathcal {P}_m ) - \dim ( p \mathcal {P}_n \cap q \mathcal {P}_m )\nonumber \\&= m+n+2 - \dim ( p \mathcal {P}_n \cap q \mathcal {P}_m ). \end{aligned}$$

Since *p* and *q* are relatively prime, any polynomial in $p \mathcal {P}_n \cap q \mathcal {P}_m$ is a multiple of *pq*. Hence, we have $p \mathcal {P}_n \cap q \mathcal {P}_m \subseteq pq \mathcal {P}_d$, where $d = \min \{ m- M, n - N\}$. This means $\dim ( p \mathcal {P}_n \cap q \mathcal {P}_m ) \le \dim \mathcal {P}_d=d+1$, and hence by (B.5) $\dim ( \mathcal {Q} ) \ge m+n+2 - ( d +1) = D + 1$. $\square$

### Lemma 4

If *r* does not belong to $\mathcal {R}_{mn}$, i.e., $M> m$ or $N> n$, then $\dim \mathcal {Q} = m+n+2$.

(Proof) This follows from (B.5) and the fact that $p \mathcal {P}_n \cap q \mathcal {P}_m = \{ 0 \}$. $\square$

We now prove results on the rank of the matrix $C_{mn}(r)$ that we use for finding the type of the rational approximant *r* in our algorithm. The first result shows that if we take *m*, *n* large enough so that $m\ge M,n\ge N$, then the rank of $C_{mn}(r)$ gives the information on $M,N$.

### Proposition 3

Assume that $$r \in \mathcal {R}_{mn}$$ is of exact type $$(M, N)$$ and $$r = p / q $$ is an irreducible expression of *r*. Then, we have

B.6 $$\begin{aligned} \mathrm {range}( C_{mn}(r) ) = \{ v( p_1 / q ) \mid p_1 \in \mathcal {P}_D \}, \end{aligned}$$

where $$D = \max \{ M+ n , m + N\}$$.

(Proof) This follows immediately from (B.4) and Lemma 3. $\square$

Note that $m\ge M,n\ge N$ is implied by the assumptions in Proposition 3. From (B.6) we see that the rank of $C_{mn}(r)$ is

B.7 $$\begin{aligned} \text{ rank }(C_{mn}(r))=D+1, \end{aligned}$$

as long as $L\ge D+1$, which always holds in our algorithm in which $L\ge m+n+1\ge D+1$. Thus we obtain $\dim \text{ null }(C) = m+n+2-(D+1)=\min (m-M,n-N)+1$, which is (3.2).

The next result shows that if we do not take *m*, *n* large enough then this is indicated by $C_{mn}(r)$ not having a null vector, provided we still sample at sufficiently many points.

### Proposition 4

Suppose that *r* of exact type $$(M, N)$$ does not belong to $$\mathcal {R}_{mn}$$, i.e., $$M> m $$ or $$N> n $$. If $$L\ge \max \{ M+ n , m + N\} + 1$$, then the rank of $$C_{mn}(r)$$ is equal to $$m+n+2$$, i.e., $$C_{mn}(r)$$ has full column rank.

(Proof) This follows from (B.4) and Lemma 4. $\square$

The above two propositions indicate that we can obtain the type $(M,N)$ of *r* by combining (1) sufficient sample points, and (2) adjusting *m*, *n* so that $C_{mn}(r)$ has null space of exactly dimension 1. This is the crux of the degree determination process described in Sect. 3.1.

### Analysis of our generalized eigenvalue problem

The above results provide information on the building-block eigenvalue problem (4.4) in terms of its regularity and eigenvalues. We say that a (possibly rectangular) matrix pencil $A-\lambda B$ is *regular* if the matrix $A-\lambda B$ has full column rank for some value of $\lambda$.

#### Proposition 5

Suppose that *f* is a rational function of exact type $(M, N)$ with $M\le m$ and $N\le n$, and $L\ge m+n+1$. If $M= m$ or $N= n$, then the matrix pencil $[A_1 , A_2] - \lambda [B_1 , O ]$ is regular and its finite eigenvalues coincide with the poles of *f*.

(Proof) The matrix pencil $[A_1 , A_2] - \lambda [B_1 , O]$ is equal to $C_{m,n-1} ( (z - \lambda ) f(z) )$.

If $\lambda$ is a pole of *f*, then $(z - \lambda ) f(z)$ is of exact type $(M, N- 1)$, and consequently, we have

$$\begin{aligned} \mathrm {rank} ( C_{m,n-1} ( (z - \lambda ) f(z) )) = \max \{ M+ n- 1 , m+ N- 1 \} + 1 = m+n\end{aligned}$$

by Proposition 3, hence $C_{m,n-1} ( (z - \lambda ) f(z) )$ is rank deficient.

Conversely, if $\lambda$ is not a pole of *f*, then $(z - \lambda ) f(z)$ is of exact type $(M+ 1 , N)$, and hence by Proposition 4, $C_{m,n-1} ( (z - \lambda ) f(z) ))$ has full column rank. $\square$

We are thus able to correctly compute the poles of *f* provided that we take one of $m,n$ to be the correct value $M,N$.

## Condition number of eigenvalues

Here we analyze the condition number of the eigenvalues of the matrix pencil $[A_1 , A_2] - \lambda [B_1 , O]$, which here we write simply as $A-\lambda B$. For simplicity we focus on the case where the sample points are roots of unity, and examine the conditioning as $n$ is fixed and the number of sample points grow along with $m$. We shall show that the eigenvalues outside the unit disk become increasingly ill-conditioned, which explains the observation in Fig. 7.

Assume that $f = p / q$ is irreducible and *f* has $N$ simple poles $\xi _1 , \ldots , \xi _{N}$. We consider the square generalized eigenvalue problem (4.4) where $n= N$ (i.e., the denominator degree is fixed to the correct value), $L=m+n+1$ and $\{ \gamma _j \}_{j=1}^L= \{ \exp ( 2 \pi \mathrm {i} \cdot j / L) \}_{j=1}^L$. For $m\ge M$, $A - \lambda B$ has eigenvalue equal to $\xi _k$ for each *k*. We will investigate how the condition number of each eigenvalue $\xi _k$ change, when we increase $m$ (and hence also $L=m+n+1$).

The absolute condition number of a simple eigenvalues of a matrix pencil $A-\lambda B$ is known [45] to be proportional to $\Vert x \Vert \Vert y \Vert / | y^\top Bx |$, where *y* and *x* are the corresponding left and right eigenvectors. Thus we need to identify the eigenvectors for $\xi _k$.

The right eigenvector $x_k$ such that $[A_1 , A_2]x_k =\xi _k [B_1 , O]x_k$ is given by $x_k = [c_{\tilde{q}}^{\top } , c_p^{\top }]^{\top }$, where $\tilde{q}(z) = q (z) /(z - \xi _k)$. This $x_k$ satisfies $B x_k = v( f \tilde{q} )$.

To find the left eigenvector, first note that as in Proposition 3 we have

$$\begin{aligned} \mathrm {range}(A - \xi _k B) = \{ v( p_1 / \tilde{q} ) \mid p_1 \in \mathcal {P}_{L-2} \}. \end{aligned}$$

Now since $\{ \gamma _j \}$ are $L$th roots of unity, the vector

$$\begin{aligned} y_k^\top = [ \tilde{q}(\gamma _1) \gamma _1, \ldots , \tilde{q} (\gamma _L) \gamma _L] \end{aligned}$$

satisfies $y_k^\top (A-\xi _kB)=0$, indicating $y_k$ is the left eigenvector.

Hence, we have

C.1 $$\begin{aligned} y_k^\top B x_k&= y_k^\top B_1 c_{\tilde{q}} = \sum _{j=1}^Lf(\gamma _j) \tilde{q}(\gamma _j) ^2 \gamma _j = \sum _{j=1}^L\frac{p(\gamma _j ) \tilde{q}(\gamma _j) ^2 \gamma _j}{q(\gamma _j )}\nonumber \\&= \sum _{j=1}^L\frac{p(\gamma _j) \tilde{q}(\gamma _j) \gamma _j}{ \gamma _j - \xi _k } . \end{aligned}$$

This implies that $y_k^\top B x_k / L$ is an approximate value of

C.2 $$\begin{aligned} I = \frac{1}{2 \pi \mathrm {i}} \int _{|z|=1} \frac{p(z)\tilde{q}(z)}{z-\xi _k} \mathrm {d}z = \left\{ \begin{array}{ll} 0 \quad &{} ( | \xi _k | > 1 ) \\ p( \xi _k ) \tilde{q} ( \xi _k ) \quad &{} ( | \xi _k | < 1 ) . \end{array} \right. \end{aligned}$$

In fact, the trapezoidal rule approximation to *I* with sample points $\{ \gamma _1 , \gamma _2 , \ldots , \gamma _L\}$ is given by

C.3 $$\begin{aligned} I_L= \frac{1}{2 \pi \mathrm {i}} \sum _{j=1}^L\frac{p(\gamma _j) \tilde{q}(\gamma _j)}{ \gamma _j - \xi _k } \frac{ 2 \pi \mathrm {i} \gamma _j }{ L} = \frac{1}{L} \sum _{j=1}^L\frac{p(\gamma _j) \tilde{q}(\gamma _j) \gamma _j}{ \gamma _j - \xi _k } = \frac{ y_k^\top B x_k }{L} . \end{aligned}$$

Now suppose that $| \xi _k | > 1$, that is, $\xi _k$ lies outside the unit disk. Then, the integrand is analytic in the disc $|z| < |\xi |$ and have a simple pole on the circle $|z| = |\xi |$, and hence the trapezoidal rule approximation satisfies (see [49] for the trapezoidal rule and its properties)

C.4 $$\begin{aligned} | I_L- I | = O ( | \xi _k |^{ - L} ). \end{aligned}$$

From (C.2), (C.3) and (C.4), we have

C.5 $$\begin{aligned} y_k^\top B x_k = O( L| \xi _k |^{ - L} ) . \end{aligned}$$

Furthermore, since $\Vert y_k \Vert = \Theta ( \sqrt{L} )$ and $\Vert x_k \Vert = {\varOmega }( 1 )$, the condition number $\kappa (\xi _k)$ of the eigenvalue $\xi _k$ satisfies

C.6 $$\begin{aligned} \kappa (\xi _k) = \frac{\Vert y_k \Vert \Vert x_k \Vert }{| y_k B x_k |} = {\varOmega } ( L^{- \frac{1}{2} } | \xi _k |^L) \quad \text{ for }\ | \xi _ k | > 1. \end{aligned}$$

This means that the condition numbers of eigenvalues outside the unit circle grow exponentially as we use more sample points.

On the other hand, if the eigenvalue $\xi _k$ is inside the unit disk, i.e., $| \xi _k | < 1$, then the condition number of $\xi _k$ behaves differently. Indeed, since we have

C.7 $$\begin{aligned} \lim _{L\rightarrow \infty } \frac{ y_k^\top B x_k }{L} = I \ne 0 , \end{aligned}$$

we see that the condition number of the eigenvalue $\xi _k$ satisfies

C.8 $$\begin{aligned} \kappa (\xi _k) = \frac{\Vert y_k \Vert \Vert x_k \Vert }{| y_k B x_k |} = O ( L^{ - \frac{1}{2} } ) \quad \text{ for }\ | \xi _ k | < 1. \end{aligned}$$

This is in sharp contrast to (C.6). Equations (C.6) and (C.8) imply that, when the number of sample points grows, the computed eigenvalues outside the unit disk lose accuracy exponentially, while those inside do not.

We confirmed this in our numerical experiments as the figures in Sect. 6 show.

## Footnotes

1 We note that these QR factorizations can be computed exploiting the Vandermonde-like structure of $DFV_{n+1},DV_{m+1}$. Namely, when the basis is degree-graded, i.e., $\deg \phi _i(z)=i$, then the column space of $DV_{m+1}$ is equal to the Krylov subspace $\mathcal {K}(\varGamma ,b)=[b,\varGamma b,\varGamma ^2b,\ldots , \varGamma ^{n-1}b]$ where *b* is the first column of $DV_{m+1}$. An orthonormal space for the Krylov subspace $\mathcal {K}(\varGamma ,b)$ can thus be computed using the Arnoldi process [19, Sect. 10.5], as done for example in [24, App. A]. The same holds for $DFV_{n+1}$.

2 When reducing the number of sample points is of primary importance (i.e., when sampling *f* is expensive), we can proceed as follows: having sampled *f* at *L* points, take $n\leftarrow n+\ell ,m\leftarrow m-\ell$ for integers $\ell$ with $|\ell |\le \frac{L}{2}$, re-form $\tilde{C}$ and examine the condition $\dim \text{ null }(\tilde{C})\ge 1$; if this is satisfied for some $\ell$, there exists an acceptable rational approximant of type $(n+\ell ,m-\ell )$.

3 For simplicity, we mainly analyze the scaled version of (4.4), without employing the projection (4.7) and QR factorization.
